# Effectiveness and treatment moderators of internet interventions for adult problem drinking: An individual patient data meta-analysis of 19 randomised controlled trials

**DOI:** 10.1371/journal.pmed.1002714

**Published:** 2018-12-18

**Authors:** Heleen Riper, Adriaan Hoogendoorn, Pim Cuijpers, Eirini Karyotaki, Nikolaos Boumparis, Adriana Mira, Gerhard Andersson, Anne H. Berman, Nicolas Bertholet, Gallus Bischof, Matthijs Blankers, Brigitte Boon, Leif Boß, Håvar Brendryen, John Cunningham, David Ebert, Anders Hansen, Reid Hester, Zarnie Khadjesari, Jeannet Kramer, Elizabeth Murray, Marloes Postel, Daniela Schulz, Kristina Sinadinovic, Brian Suffoletto, Christopher Sundström, Hein de Vries, Paul Wallace, Reinout W. Wiers, Johannes H. Smit

**Affiliations:** 1 Department of Clinical, Neuro- and Developmental Psychology, VU University, Amsterdam, the Netherlands; 2 Department of Psychiatry, VU University Medical Centre, Amsterdam, the Netherlands; 3 APH Institute for Health and Care Research, VU University Medical Centre, Amsterdam, the Netherlands; 4 Department of Psychology and Technology, Universitat Jaume I, Castellon, Spain; 5 Department of Psychology and Sociology, Universidad de Zaragoza, Zaragoza, Spain; 6 Department of Behavioural Sciences and Learning, Linköping University, Linköping, Sweden; 7 Centre for Psychiatry Research, Department of Clinical Neuroscience, Karolinska Institutet, Stockholm, Sweden; 8 Stockholm Health Care Services, Stockholm County Council, Stockholm, Sweden; 9 Alcohol Treatment Centre, Department of Community Medicine and Health, Lausanne University Hospital, Lausanne, Switzerland; 10 Department of Psychiatry and Psychotherapy, University of Lübeck, Lübeck, Germany; 11 Arkin Mental Health Care, Department of Research, Amsterdam, the Netherlands; 12 Academic Medical Centre, Department of Psychiatry, University of Amsterdam, Amsterdam, the Netherlands; 13 Trimbos Institute—Netherlands Institute of Mental Health and Addiction, Utrecht, the Netherlands; 14 Academy Het Dorp, Arnhem, the Netherlands; 15 Department of Health Psychology and Applied Biological Psychology, Institute of Psychology, Leuphana University of Lüneburg, Lüneburg, Germany; 16 Norwegian Centre for Addiction Research, Institute of Clinical Medicine, Faculty of Medicine, University of Oslo, Oslo, Norway; 17 Centre for Addiction and Mental Health, University of Toronto, Ontario, Canada Department of Psychiatry and Department of Psychology, University of Toronto, Toronto, Ontario, Canada; 18 Centre for Addiction and Mental Health, Toronto, Ontario, Canada; 19 Research School of Population Health, Australian National University, Canberra, Australia; 20 Department of Clinical Psychology and Psychotherapy, Friedrich-Alexander-Universität Erlangen-Nürnberg, Erlangen, Germany; 21 Strategic Research and Development Support, Metropolitan University College, Copenhagen, Denmark; 22 Research Division, Behavior Therapy Associates, Albuquerque, New Mexico, United States of America; 23 School of Health Sciences, University of East Anglia, Norwich, United Kingdom; 24 Health Service and Population Research Department, Centre for Implementation Science, Institute of Psychiatry, Psychology and Neuroscience, King’s College London, London, United Kingdom; 25 eHealth Unit, Research Department of Primary Care and Population Health, University College London, Royal Free Hospital, London, United Kingdom; 26 Department of Psychology, Health and Technology, University of Twente, Enschede, the Netherlands; 27 Department of Development and Advice, Tactus Addiction Treatment, Deventer, the Netherlands; 28 CAPHRI School for Public Health and Primary Care, Department of Health Promotion, Maastricht University, Maastricht, the Netherlands; 29 Department of Emergency Medicine, University of Pittsburgh, Pittsburgh, Pennsylvania, United States of America; 30 Institute for Mental Health Policy Research, Centre for Addiction and Mental Health, Toronto, Ontario, Canada; 31 Addiction Development and Psychopathology (ADAPT) Laboratory, Department of Psychology, University of Amsterdam, the Netherlands; University of New South Wales, AUSTRALIA

## Abstract

**Background:**

Face-to-face brief interventions for problem drinking are effective, but they have found limited implementation in routine care and the community. Internet-based interventions could overcome this treatment gap. We investigated effectiveness and moderators of treatment outcomes in internet-based interventions for adult problem drinking (iAIs).

**Methods and findings:**

Systematic searches were performed in medical and psychological databases to 31 December 2016. A one-stage individual patient data meta-analysis (IPDMA) was conducted with a linear mixed model complete-case approach, using baseline and first follow-up data. The primary outcome measure was mean weekly alcohol consumption in standard units (SUs, 10 grams of ethanol). Secondary outcome was treatment response (TR), defined as less than 14/21 SUs for women/men weekly. Putative participant, intervention, and study moderators were included. Robustness was verified in three sensitivity analyses: a two-stage IPDMA, a one-stage IPDMA using multiple imputation, and a missing-not-at-random (MNAR) analysis. We obtained baseline data for 14,198 adult participants (19 randomised controlled trials [RCTs], mean age 40.7 [SD = 13.2], 47.6% women). Their baseline mean weekly alcohol consumption was 38.1 SUs (SD = 26.9). Most were regular problem drinkers (80.1%, SUs 44.7, SD = 26.4) and 19.9% (SUs 11.9, SD = 4.1) were binge-only drinkers. About one third were heavy drinkers, meaning that women/men consumed, respectively, more than 35/50 SUs of alcohol at baseline (34.2%, SUs 65.9, SD = 27.1). Post-intervention data were available for 8,095 participants. Compared with controls, iAI participants showed a greater mean weekly decrease at follow-up of 5.02 SUs (95% CI −7.57 to −2.48, *p* < 0.001) and a higher rate of TR (odds ratio [OR] 2.20, 95% CI 1.63–2.95, *p* < 0.001, number needed to treat [NNT] = 4.15, 95% CI 3.06–6.62). Persons above age 55 showed higher TR than their younger counterparts (OR = 1.66, 95% CI 1.21–2.27, *p* = 0.002). Drinking profiles were not significantly associated with treatment outcomes. Human-supported interventions were superior to fully automated ones on both outcome measures (comparative reduction: −6.78 SUs, 95% CI −12.11 to −1.45, *p* = 0.013; TR: OR = 2.23, 95% CI 1.22–4.08, *p* = 0.009). Participants treated in iAIs based on personalised normative feedback (PNF) alone were significantly less likely to sustain low-risk drinking at follow-up than those in iAIs based on integrated therapeutic principles (OR = 0.52, 95% CI 0.29–0.93, *p* = 0.029). The use of waitlist control in RCTs was associated with significantly better treatment outcomes than the use of other types of control (comparative reduction: −9.27 SUs, 95% CI −13.97 to −4.57, *p* < 0.001; TR: OR = 3.74, 95% CI 2.13–6.53, *p* < 0.001). The overall quality of the RCTs was high; a major limitation included high study dropout (43%). Sensitivity analyses confirmed the robustness of our primary analyses.

**Conclusion:**

To our knowledge, this is the first IPDMA on internet-based interventions that has shown them to be effective in curbing various patterns of adult problem drinking in both community and healthcare settings. Waitlist control may be conducive to inflation of treatment outcomes.

## Abstract 2

In this meta-analysis using individual patient data from 19 randomised controlled trials, Heleen Riper and colleagues investigate which internet-based interventions for decreasing problem drinking work best and how participant and intervention characteristics moderate the effectiveness of these interventions.

## Author summary

**Why was this study done?:**

- Global estimations continue to show increasing morbidity, mortality, and social harm caused by all types of problem drinking.
- Face-to-face brief interventions for problem drinking are effective but rarely used.
- Internet-based interventions could overcome this treatment gap.
- We investigated effectiveness and moderators of treatment outcomes in internet-based interventions for adult problem drinking.

**What did the researchers do and find?:**

- We conducted a one-stage individual patient data meta-analysis (IPDMA). This is, to our knowledge, the first study to identify moderators at the participant, intervention, and study design levels that are associated with treatment outcomes in internet-based interventions for adult problem drinking.
- Our IPDMA included 14,198 adults at baseline from 19 randomised controlled trials who exhibited various profiles of problem drinking. We obtained posttreatment data for 8,095 participants.
- Our results show that internet-based alcohol interventions in both community and healthcare populations are effective in reducing mean weekly alcohol consumption and in achieving adherence to low-risk drinking limits.
- We did not find differences in impact related to drinking profiles, meaning that people exceeding risk limits to a smaller or a larger degree benefited from the interventions, as did binge-only drinkers. Human-guided interventions showed a stronger impact on treatment outcome than fully automated ones, but waitlist design controls may inflate outcomes.

**What do these findings mean?:**

- The health gains of internet-based alcohol interventions could be substantial, because such programmes can reach high numbers of problem drinkers by virtue of their swift entry procedures and their easy scalability.
- Future research should seek to identify categories of people for whom such interventions work best, to analyse how the interventions work and to determine what delivery contexts are most favourable. It should explore which patient populations could benefit most from referral to unguided forms and which would be more amenable to guidance by GPs or other professionals.

## Introduction

Global estimations continue to show increasing physical and psychological morbidity, all-cause and specific-cause mortality, and social harm deriving from all types of alcohol misuse. Usually, a positive and linear association is seen between increased consumption and related health risks [1]. A number of factors underlie this mounting health burden. These include increases in the prevalence of alcohol consumers due to population growth and societal ageing, an absolute increase in adult alcohol consumption due to greater wealth and wider acceptance of alcohol use, and escalating alcohol use amongst women and the elderly. At the same time, there are growing insights into health risks connected with even minimal levels of alcohol consumption [2,3].

Brief alcohol interventions (BAIs) in primary care and community settings have been found clinically and cost-effective, with effect sizes in the small to moderate range, for reducing both hazardous drinking (which increases the risk of physical or psychological harm) and harmful drinking (which has already caused some damage) [4]. Together, their target groups are referred to as ‘problem drinkers’ to distinguish them from drinkers with alcohol use disorders, for whom more intensive treatments are recommended [5]. Problem drinkers account for the highest prevalence of alcohol misuse. Based on accumulated evidence, many national and professional guidelines now recommend brief interventions for problem drinkers in primary care settings and among community populations [6]. These interventions are comprised mostly of brief single or multiple sessions (up to six) and are based on personalised normative feedback (PNF) [7] or combinations of PNF, motivational interviewing (MI) [8], cognitive-behavioural therapy (CBT) [9], or behavioural self-control (BSC) principles [10]. Despite the ample evidence available, the actual impact of BAIs on curbing the prevalence of problem drinking in the wider population has been disappointingly low. The main factors in the weak impact include problems with implementation, as relatively few healthcare professionals actually administer BAIs; in addition, only a small proportion of patients who might benefit are actually offered BAIs, and even fewer accept the offer [11].

Internet-based alcohol interventions (iAIs) may overcome some of these problems by virtue of their low-threshold accessibility, their high scalability, and their acceptability to problem drinkers, as was recently echoed by McCambridge and Saitz [11]. Major advantages of iAIs, as perceived by many problem drinkers, are reduced stigma and greater comfort about disclosing drinking problems. The majority of iAIs are based on manualised therapeutic principles similar to those in BAIs. They are offered in unguided and guided formats. Unguided iAIs are fully automated interventions that participants can perform without human guidance. Guided interventions provide human support to guide participants through the intervention, mainly via asynchronous secure email contact [12]. The support may come from health professionals or trained volunteers. Meta-analytic studies have shown that unguided iAIs, in particular, are now used on a wider scale than conventional BAIs [13]. They have been found clinically effective (small effects) in reducing mean weekly adult alcohol consumption as compared with controls [14]. As a result, iAIs have been incorporated into some clinical guidelines for treating problem drinking in primary care [15].

All this notwithstanding, various uncertainties still surround the evidence base for iAIs. First of all, still little is known about whether women and older people derive benefits comparable to those seen for male and younger problem drinkers. Such knowledge is important in view of the rising prevalence rates of problem drinking among women and the elderly and their underrepresentation in many intervention studies [16]. Secondly, problem drinking actually embraces several different drinking profiles, and only a few iAI studies have investigated whether these might moderate treatment outcomes [17]. Such profiles include exceeding the advised weekly alcohol limits to a moderate (‘regular drinking’) or a serious degree (‘heavy drinking’) and ‘binge-only drinking’, whereby alcohol users episodically exceed the maximum advised intakes per drinking occasion. Such divergent drinking profiles may or may not necessitate different interventions. Thirdly, there is the question of whether guided iAIs are more effective than unguided ones—a finding reported for CBT-based internet interventions for common mental disorders such as depression [18]. A related question is whether iAI treatment outcomes might vary according to the therapeutic orientation of the intervention.

The few moderator analyses conducted to date had a common limitation: they were statistically underpowered to properly address such questions [14]. To overcome this major problem, we conducted an individual patient data meta-analysis (IPDMA) that boosted the number of participants studied and thereby the statistical power. That enabled us to better evaluate the overall effectiveness of iAIs in reducing alcohol consumption, as well as to explore statistically significant differences within the data by performing moderator analyses on treatment outcomes, with a focus on participant, intervention, and study design characteristics.

## Materials and methods

### Identification and selection of randomised controlled trials

PsycINFO, Science Citation Index Expanded, Social Sciences Citation Index, Arts and Humanities Citation Index, CINAHL, PubMed, and EMBASE were searched up to 31 December 2016. All papers retrieved were evaluated by independent assessors (HR, EK, or NBo) (for search string, see S1 Data).

### Eligibility criteria

Randomised controlled trials (RCTs) were eligible if they (1) studied people aged ≥18 with quantifiable levels of alcohol consumption that exceeded recommendations for low-risk drinking; (2) compared an iAI with a control condition (e.g., assessment only, waitlist, or minimal intervention); (3) studied an iAI based on therapeutic principles such as PNF, BSC, CBT, MI, or combinations thereof; and (4) studied either an unguided or a guided intervention or both. RCTs in populations of students or pregnant women were excluded. Primary authors of identified trials were asked to provide their raw RCT data for a set of pre-identified variables (HR/EK, S2 Data and see S4 Data for data access contact list of original studies) and were queried as to whether they were aware of ongoing RCTs that met our inclusion criteria; two more RCTs were thus identified [19,20]. No study protocol for this study has been developed.

### Risk-of-bias assessment and data extraction

Five criteria from the Cochrane Collaboration risk-of-bias assessment tool were applied (by EK, HR, and NBo): (1) adequate random sequence allocation, (2) concealment of allocation to the different conditions, (3) blinding of participants and therapists to the study condition, (4) blinding of assessors to outcomes, and (5) handling of missing data [21].

### IPDMA

#### Primary outcome measure

The primary outcome was mean weekly alcohol consumption, expressed in standard units. As RCTs differ in the quantification of alcohol in beverages, based on national custom (ranging from 8 to 14 grams of ethanol per unit [22]), we recalculated these into standard units of alcohol consumption based on 10 grams of ethanol (SUs). Most RCTs measured alcohol consumption using time line follow-back (TLFB) approaches. For a few RCTs that did not report TLFB data, we estimated mean weekly SUs on the basis of the first two questions of The Alcohol Use Disorders Identification Test (brief, 3 items; AUDIT-C) scale [23] at post-intervention. Alcohol consumption at baseline was constructed identically. Most included participants were regular drinkers who were consuming more than the recommended low-risk weekly limits of 14 SUs (females) or 21 SUs (males) at baseline. Binge-only drinking is another problem drinking profile in which low-risk recommendations are exceeded. We defined binge-only drinkers by proxy as participants who drank more than 4 or 6 SUs (females/males) on at least one occasion per week, while still totalling less than 14/21 SUs weekly.

#### Secondary outcome measure

Treatment response (TR) was defined as an alcohol consumption level below 14/21 SUs per week for females/males at the first post-intervention follow-up.

#### Moderators

The following participant-level putative moderators were tested: gender (female/male); age (below 55/above 55); education (high/low, with ‘high’ referring to tertiary education and ‘low’ to primary or secondary schooling), employment (yes/no), and partner relationship (yes/no). Two dimensions of problem drinking were explored: regular drinking (>14/21 SUs female/male weekly) as contrasted with binge-only drinking (≥4/6 SUs female/male at least once a week but below 14/21 female/male SUs weekly); and heavy drinking (>35/50 SUs female/male weekly) as contrasted with non-heavy drinking (14–35 SUs weekly in females and 21–50 SUs weekly in males). Intervention-level putative moderators were therapeutic guidance (human-guided versus unguided interventions), intensity (single versus multiple sessions), therapeutic orientation (PNF-only versus integrated therapeutic principles), and intervention setting (in work, healthcare, or community populations). A study design moderator, type of control, was also included (waitlist control contrasted with assessment-only or minimal-intervention control).

#### One-stage and two-stage IPDMAs

Replications of individual study outcomes based on the raw data in comparison with the published results led to only one correction to the published tables [24]. We next applied a one-stage individual patient data (IPD) model of analysis, as it is assumed to produce a more exact likelihood specification than a two-stage approach [25]. In a one-stage IPDMA, the effect of iAIs is evaluated by fitting a single comprehensive model to the IPD from all trials, while simultaneously accounting for the nesting of participants within these trials. To account for the nesting structure, we assessed the summary effect of iAIs on the primary outcome using a linear mixed model (LMM). At the participant level, we used an ANCOVA model [26], regressing the post-intervention outcome score on the iAI intervention indicator, with the baseline alcohol consumption score used as a covariate.

To deal with missing baseline alcohol data, we used mean imputation to estimate scores [27]. We subsequently analysed all available outcomes using complete cases—that is, including the full baseline outcomes (*N* = 14,198) but ignoring missing post-intervention outcomes. This analysis implicitly assumes that the missing data are missing at random (MAR) rather than missing completely at random (MCAR), allowing missingness of post-intervention scores to depend on the pre-intervention score.

To evaluate the effect of iAIs using an LMM, we regressed the post-intervention weekly SU level on the iAI intervention indicator, the baseline weekly SU level, and the comparison indicators (dummy variables contrasting the intervention arms with the control arms of the trials), assuming random effects (both intercepts and intervention slopes) for those comparisons and equal residual variances across trials. The estimates of the iAIs’ effects are presented as unstandardised regression coefficients (*b*), which refer to the overall effect of the intervention on posttreatment drinking behaviour in terms of comparative SU levels.

For TR, a generalised LMM with participants nested within trials (a logistic model) was similarly used. TR at follow-up (yes/no) was the dichotomous dependent variable, and all fixed and random effects were identical to those in the LMM for the continuous primary outcome, except that fixed intercepts were removed for reasons of identification (convergence), resulting in a model with random intercepts (and slopes). We calculated odds ratios (ORs), representing the probability that an outcome will occur given a particular exposure as compared with the probability in the absence of that exposure [28]. TR was additionally interpreted by transforming the OR to a number needed to treat (NNT) [29].

We subsequently tested whether participant, intervention, or study design characteristics moderated the effect of iAIs on either the primary or the secondary outcome or both. However, participant-level characteristics and study- and intervention-level characteristics were analysed differently. For participant-level characteristics, within-study and across-study interaction effects had to be separated to avoid ecological bias [25], whilst no such separation was needed for study- and intervention-level moderators. We additionally performed two-stage analyses to evaluate the sensitivity of the one-stage results, a recommendation by Burke and colleagues [25]. The two-stage approach in our study derived aggregate data for effect estimates and their CIs for each study individually (step one), then combined these in a conventional meta-analysis model (step two). In two-stage analyses, participant-level moderators are estimated for each study separately and combined in the second stage, without risk of ecological bias, while intervention- and study-level characteristics are studied by comparing subgroups of trials in the second stage.

In the one-stage approach, we added a second sensitivity analysis to compare our results against those of a procedure applying multiple imputation to include all participants (an intention-to-treat [ITT] analysis). This analysis was conducted on the request of one of the reviewers. The multiple imputation procedure used chained equations to impute missing alcohol consumption scores—both before and after the intervention—together with missing values of the participant-level putative moderators. The ITT analysis employed logistic regression models for the dichotomous variables and predicted-mean matching for the continuous variables, with study indicators and intervention indicators (fully interacted) included as covariates. This second sensitivity analysis tested the main intervention effect and the moderator effects of all participant-level and study-level moderators on the primary outcome variable.

In the two-stage approach, we employed a third sensitivity analysis that evaluated the MAR assumption on the missing data mechanism, thereby answering an additional question of one of the reviewers. This additional, missing-not-at-random (MNAR) analysis is part of the ITT strategy suggested by White and colleagues [30]; it includes all randomised individuals in the analysis, taking baseline outcomes of dropouts into account. It evaluates a series of values of a sensitivity parameter δ, which equals the difference between the mean of the observed values of post-intervention SUs of alcohol and the mean of the unobserved values. Under MAR, δ (being the covariate-adjusted mean difference between missing and observed outcomes) is assumed to be zero. In our case, positive (or negative) values of δ correspond to the situation in which the dropouts, after adjustment for pre-intervention SUs, would have higher (or lower) mean values of post-intervention SUs than those who continued participation (see S1 Text for a detailed explanation of the evaluation of the MAR assumption). This third sensitivity analysis targeted the main intervention effect on the primary outcome variable only.

To examine heterogeneity, we calculated the *I*^*2*^ statistic using the two-stage approach as well. This indicator is expressed as a percentage: an *I*^*2*^ value of 0% is interpreted as no heterogeneity, 25% as low, 50% as moderate, and 75% as high heterogeneity [31]. We calculated the 95% CIs around *I*^*2*^ using the noncentral chi-squared–based approach within the heterogeneity module for Stata [32,33]. All analyses were conducted with Stata 14.2.

### Comparison of IPDMA-included with non-included RCTs

The potential differences in treatment outcomes between the trials included and those that could not be included in preparing our IPDMA were assessed with a conventional meta-analysis (Comprehensive Meta-Analysis, version 3.3.070; S3 Data).

## Results

Results are reported in accordance with the Preferred Reporting Items for Systematic Review and Meta-Analyses for IPD (S1 PRISMA Checklist) [34].

### Selection of RCTs

**Fig 1:**
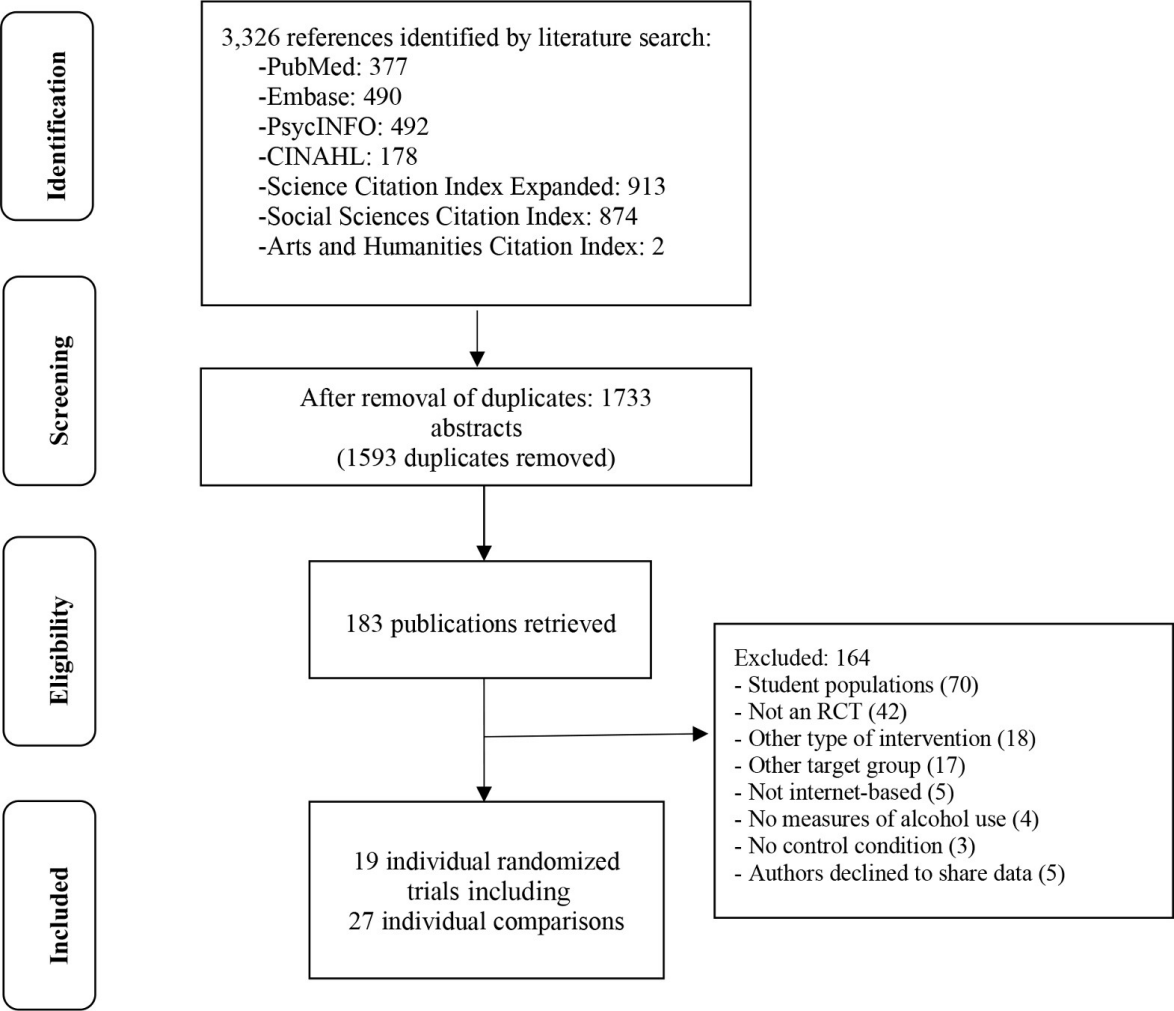
PRISMA IPD diagram of RCTs selection process. IPD, individual patient data; RCT, randomised controlled trial.

Fig 1 illustrates the selection process for the trials included in our IPDMA. We identified 183 full papers, from which 24 eligible RCTS were found, five of which [35–39] could not be included (all involving unguided iAIs) because authors did not respond to our invitation.

### Study characteristics

Table 1 shows the characteristics of the 19 included RCTs (26 comparisons). Most trials applied the full Alcohol Use Disorders Identification Test (AUDIT) (*n* = 9, cutoff ≥8) [40] or AUDIT-C scales (*n* = 4, cutoff ≥4 or ≥5) [23] as inclusion criteria. Four RCTs used cutoff thresholds based on daily or weekly low-risk drinking recommendations; the Fast Alcohol Screening Test (FAST) was applied in two trials [41]. Participants were recruited either directly from the community (*n* = 12 trials), from healthcare settings (*n* = 4), or from work settings (*n* = 3). Eight trials employed a minimal-intervention control design, six trials applied assessment-only control, and five included a waitlist-control comparator. Eleven trials estimated the effects of multiple-session iAIs, seven studied single-session iAIs, and one study included both types. Twelve investigated effects of therapeutically integrated iAIs and seven studied PNF-only interventions. Most comparisons (*n* = 19) involved unguided iAIs; eight involved human-guided interventions. The first post-intervention assessment occurred in most trials (*n* = 15) between 1 and 3 months after treatment, in three trials at 6 months, and in one study at 12 months. A total of *N* = 14,198 participants was included, out of the 17,545 participants in the 24 identified trials (a 79.77% inclusion rate).

**Table 1:** Characteristics of studies analysed in IPDMA (19 studies, 27 comparisons).

Study	Target group/Screener	Setting/Recruitment	Intervention	Mode of delivery	*N*	Control	Timing of FPTA
Araki et al. 2006 / JP [42]	Males >20 g ethanol/day, abnormal serum γ-GT	Workplace	Alcohol psychoeducation—MSG	Email	24	Waitlist	2 months
Bertholet et al. 2015 / CH [43]	Males age 21, AUDIT score >8 or >140 g ethanol/week; ≥60 g/occasion	General population cohort, army conscription centres	PNF extended—SSU	Internet	737	Assessment-only	1 month
Bischof et al. 2008 / DE [44]	AUDIT >5, LAST ≥2	General practices	a) TTM/BCC/MI: SC: digital + brief phone feedback—MSG	Digital + phone	408	Health behaviour booklet	12 months
			b) TTM/BCC/MI: Full: digital + longer phone feedback—MSG				
Blankers et al. 2011 / NL [45]	AUDIT >8 and >140 g ethanol/week	Community and SATC	a) CBT/MI—MSU	Internet	205	Waitlist	3 months
			b) CBT/MI—MSG				
Boon et al. 2011 / NL [46]	Males >200 g ethanol/week and/or >50 g ethanol on ≥1 day/week	Community	PNF—SSU	Internet	450	Alcohol leaflet	1 month
Boß et al. (2017) / DE [47]	F/M AUDIT score >6/8 and F/M >140/210 g ethanol/week	Community	a) PNF/MI/BA—MSG	Internet	432	Waitlist	1.5 months
			b) PNF/MI/BA—MSU				
Brendryen et al. 2014 / NO [48]	FAST ≥3	Community	PNF/BSC/CBT—MSU	Internet	244	PNF/SSU/e-booklet	2 months
Brendryen et al. 2017 / NO [20]	FAST ≥3	Workplace	PNF/ /BSC/CBT—MSU	Internet	85	PNF/SSU/e-booklet	2 months
Cunningham et al. 2009 / CA [49]	AUDIT-C ≥4	Community	PNF—SSU	Internet	72	Alcohol leaflet	3 months
Hansen et al. 2012 / DK [50]	F/M >140/210 g ethanol/week	Community	PNF—SSUPBA—SSU	Internet	761	Assessment-only	6 months
Hester et al. 2005 / US [51]	AUDIT ≥8	Community	PNF/BSC/MI—SSU	CD-ROM; healthcare setting	61	Waitlist	1 month
Khadjesari et al. 2014 / UK [52]	AUDIT-C ≥5	Workplace	PNF/SC—SSU	Internet	1,330	Assessment-only	3 months
Postel et al. 2010 / NL [53]	F/M ≥150/220 g ethanol/week with upper limit of F/M <670/990 g	Community and SATC	CBT/MI—MSG	Internet	156	Waitlist	3 months
Riper et al. 2008 / NL [54]	F/M >140/210 g ethanol/week or F/M ≥40/60 g ethanol/≥1 day in past 3 months	Community	CBT/BSC/MI—MSU	Internet	261	e-Alcohol leaflet	6 months
Schulz et al. 2013 / DE [55]	AUDIT >7^1^ or F/M >10/20 g ethanol/day or drinking >5 days/week	Community	Alternating versus summative PNF—MSU	Internet	498	Assessment-only	6 months
Sinadinovic et al. 2014 / SE [56]	F/M AUDIT ≥6/8	Community	PNF/CBT/MI—MSU	Internet	633	Assessment-only	3 months
			PNF/CBT/MI—SSU				
Suffoletto et al. 2012 / US [57]	F/M AUDIT-C ≥3/4 in past 3 months	Emergency department	PNF—MSU with monitoring only PNF—MSU	SMS	45	Assessment-only	3 months
Sundström et al. 2016 / SE [58]	F/M AUDIT ≥6/8	Community/web	a) CBT/BSC/MI—MSG asynchronous	Internet	80	Web-based unguided self-help	2.5 months
			b) CBT/BSC/MI—MSG synchronous				
Wallace et al. 2011 / UK [59]	AUDIT-C ≥5	Community/web	CBT/BSC/MI—MSU	Internet	1,281^2^	Unguided noninteractive web psychoeducation	3 months

^1^Only participants scoring ≥8 on AUDIT were included in IPDMA.

^2^Main trial.

Abbreviations: γ-GT, gamma-glutamyltransferase; AA, alcohol abuse; AD, alcohol dependence; AR, at-risk drinking; AUDIT, Alcohol Use Disorders Identification Test; AUDIT-C, Alcohol Use Disorders Identification Test–Consumption; BA, behavioural activation; BCC, behavioural change counselling; BSC, behavioural self-control training; CA, Canada; CBT, cognitive-behavioural therapy; CD-ROM, Compact Disc, read-only-memory; CH, Switzerland; DE, Germany; DK, Denmark; FAST, Fast Alcohol Screening Test (≥3 indicates problem drinking); F/M, female/male; FPTA, first posttreatment assessment; G, human-guided; HDE, heavy drinking episode; IPDMA, individual patient data meta-analysis; JP, Japan; LAST, Luebeck Alcohol Dependence and Abuse Screening Test; M-CIDI, Munich Composite International Diagnostic Interview; MI, motivational interviewing; MSG, multiple-session guided; MSU, multiple-session unguided; NL, Netherlands; NO, Norway; PBA, personalised brief advice intervention; PNF, personalised normative feedback; SATC, substance abuse treatment centre; SC, stepped care; SE, Sweden; SMS, Short Message Service; SSU, single-session unguided; TTM, Transtheoretical Model of Behavioral Change; UK, United Kingdom; US, United States; web, world wide web.

### Participants’ characteristics at baseline

Of the total of 14,198 enrolled participants, 8,095 provided post-intervention outcome data (complete cases, Table 2). The mean age of the overall sample was 40.7 (SD = 13.2) and the sample was rather evenly divided by gender (47.6% women, 52.4% men). Some 51.9% of participants had tertiary education, 74.8% had paid employment, and 56.7% were in partner relationships. The mean weekly SU level at baseline was 38.1 (SD = 26.9). Most problem drinkers (80.1%, SUs 44.7, SD = 26.4) could be categorised as regular drinkers and 19.9% (SUs 11.9, SD = 4.1) as binge-only drinkers. Regular drinkers could be distinguished into heavy drinkers (34.2%, SUs 65.9, SD = 27.1) and non-heavy drinkers (65.8%, SUs 23.7, SD = 10.6). Heavy drinkers were found in both unguided and guided iAIs (34% and 30%, respectively). The mean full AUDIT score (*n* = 9 trials) was 15.0 (SD = 6.8), indicating hazardous or harmful alcohol use [40]. Of the participants for which a full AUDIT score was available, 22.2% (*n* = 678) scored above 20, indicating a risk of alcohol dependence. Missing SU scores at baseline were virtually nil (0.4%). Missing data at the first post-intervention assessment for the primary outcome were considerable (43%), predominantly resulting from study dropout, which was not entirely random: participants under age 55 and those with baseline heavy-drinking profiles dropped out significantly more than others.

**Table 2:** Characteristics of all study participants at baseline (*n =* 14,198) and complete cases (*n =* 8,095).

Baseline participant characteristics	All respondents			Complete cases		
	Males	Females	Total	Males	Females	Total
	7,443	6,755	14,198	4,437	3,658	8,095
	(52.4%)	(47.6%)	(100%)	(54.8%)	(45.2%)	(100%)
Age (SD)	41.0	40.4	40.7	41.6	41.7	41.6
	(14.1)	(12.2)	(13.2)	(14.8)	(12.4)	(13.8)
Education** tertiary (yes)	2,785/5,440	3,095/5,895	5,880/11,335	1,520/2,901	1,719/3,117	3,239/6,018
	51.2%	52.5%	51.9%	52.4%	55.1%	53.8%
Employed (yes)**	2,095/2,774	1,203/1,634	3,298/4,408	1,557/2,034	859/1,145	2,416/3,179
	75.5%	73.6%	74.8%	76.5%	75.0%	76.0%
Partner (yes)**	3,567/5,985	3,222/5,999	6,789/11,984	2,051/3,314	1,742/3,182	3,793/6,496
	59.6%	53.7%	56.7%	61.9%	54.7%	58.3%
SU/week at baseline (SD)	41.0	35.0	38.1	35.8	32.1	34.1
	(29.9)	(22.8)	(26.9)	(27.2)	(20.9)	(24.6)
**Problem-drinking profiles**						
***Patterns***						
Regular drinking*	5,488	5,878	11,366	2,970	3,112	6,082
	73.7%	87.0%	80.1%	66.9%	85.1%	75.1%
SU (SD)^1^	51.0	38.8	44.7	47.2	36.1	41.5
	(28.7)	(22.1)	(26.2)	(26.5)	(20.2)	(24.1)
Binge-only drinking	1,955	877	2,832	1,467	546	2,013
	26.3%	13.0%	19.9%	33.1%	14.9%	24.9%
SU (SD)^2^	13.0	9.5	11.9	12.8	9.5	11.9
	(4.1)	(2.8)	(4.1)	(4.1)	(2.8)	(4.1)
***Quantities***						
Heavy drinkers	2,142	2,707	4,849	977	1,283	2,260
	28.8%	40.1%	34.2%	22.0%	35.1%	27.9%
SU (SD)	78.6	55.9	65.9	76.4	53.7	63.5
	(27.7)	(21.9)	(27.1)	(27.2)	(20.6)	(26.2)
Non-heavy drinkers	5,301	4,048	9,349	3,460	2,375	5,835
	71.2%	59.9%	65.8%	78.0%	64.9%	72.1%
SU (SD)	25.8	21.0	23.7	24.3	20.5	22.8
	(11.9)	(7.9)	(10.6)	(11.8)	(7.8)	(10.6)
AUDIT	14.2	16.6	15.0	13.4	15.6	14.1
(SD)	(6.6)	(6.7)	(6.8)	(6.2)	(6.4)	(6.4)
(*n* of studies)	9	7	9	8	7	8
(*n* of participants)	2,027	975	3,002	1,625	658	2,283
***Dropout***						
All	3,006	3,097	6,103			
	40.4%	45.8%	43.0%			
Age ≥ 55	460/1,460	321/881	781/2,341			
	31.5%	36.4%	33.4%			
Binge-only drinkers	488/1,955	331/877	819/2,832			
	25.0%	37.7%	28.9%			
Heavy drinkers	1,165/2,142	1,424/2,707	2,589/4,849			
	54.4%	52.6%	53.4%			
Follow-up data available	4,437	3,658	8,095			

*Outliers above 175 SUs per week were set at 175 SUs (*n* = 23 participants at baseline).

**These variables were not assessed in all studies, therefore the numerator and denominator are listed here separately.

^1^Regular drinking denotes 14 or more SUs weekly for females or 21 or more for males (thus excluding binge-only drinking).

^2^Binge-only drinking denotes more than 4 or 6 SUs (females/males) on at least one occasion per week, while still totalling less than 14/21 SUs weekly.

Abbreviations: AUDIT, The Alcohol Use Disorders Identification Test; SU, standard unit of alcohol consumption based on 10 grams of ethanol.

### Risk of bias

**Fig 2:**
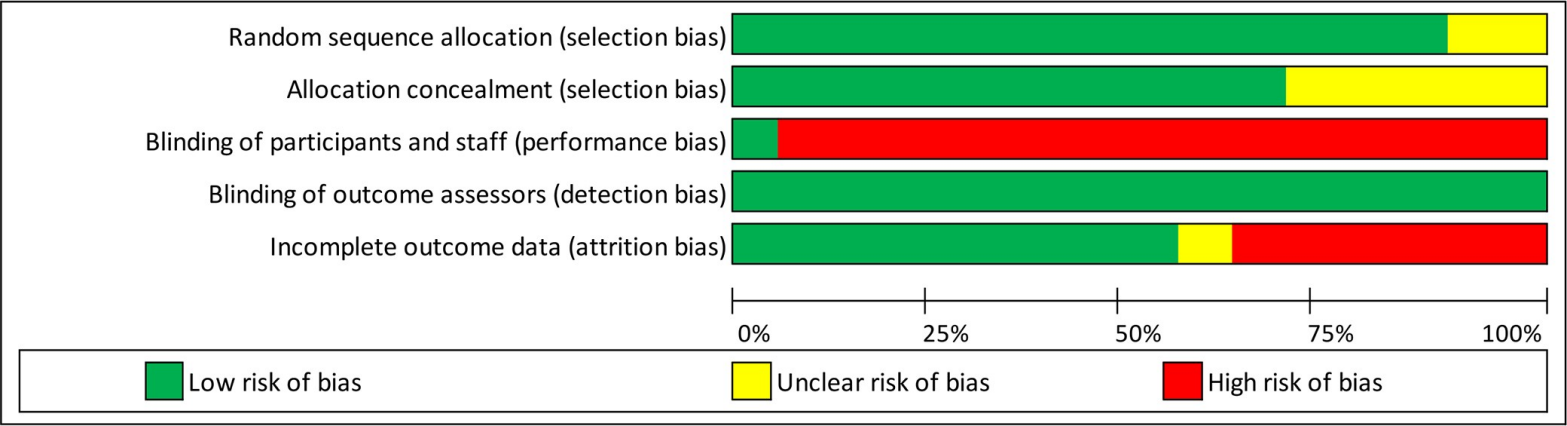
Risk-of-bias assessment.

The quality of the RCTs was relatively high (Fig 2 and S1 Table). All but one scored high-risk on the blinding of participants, which was expected, as this criterion is difficult to meet for behavioural change trials. All trials included ITT analyses, but seven had a high bias risk in terms of high study dropout (over 30%).

### One-stage IPDMA analyses: Main outcomes

The overall difference in mean weekly alcohol reduction was significant and in favour of the iAI condition (*b* = −5.02 SUs, 95% CI −7.57 to −2.48, *p* < 0.001; see Table 3). We identified four outliers (RCTs in which the 95% CI did not overlap with that of our pooled effect size) [43,52,53,59]. Removal of these outliers altered the result only slightly (*b* = −4.81 SUs, 95% CI −6.69 to −2.93, *p* < 0.001). For two trials (Khadjesari 2014, *N* = 1,330, and Sinadinovic 2014, *N* = 633), we had estimated the mean weekly SUs on the basis of the first two questions of the AUDIT-C; removal of those trials likewise only slightly altered the result (*b* = −5.74 SUs, 95% CI −8.55 to −2.92, *p* < 0.001).

**Table 3:** Effects of iAIs in terms of weekly SUs, moderating effects, and subgroup analyses (one- and two-stage results).

Primary outcome (SUs) and outcome moderators	Comparisons	Persons^5^	One-Stage Results			Two-Stage Results		
			Effect^6^	95% CI	*p*-value	Effect^6^	95% CI	*p*-value
***Overall effect***	**27**	**8,095**	***b* = −5.02 SU**	**(−7.57, −2.48)**	**<0.001**	*b =* −4.80 SU	(−6.99, −2.61)	**<0.001**
With outlier studies removed^1^	**23**	**3,129**	***b* = −4.81 SU**	**(−6.69, −2.93)**	**<0.001**	*b =* −4.36 SU	(−5.93, −2.78)	**<0.001**
With studies with AUDIT-only estimates removed^2^	**24**	**6,909**	***b* = −5.74 SU**	**(−8.55, −2.92)**	**<0.001**	*b =* −5.54 SU	(−7.99, −3.09)	**<0.001**
***Participant-level characteristics***								
***Sociodemographics***								
Gender (female)	**27**	**8,095**	***b* = 2.19 SU**	**(0.52, 3.85)**	**0.010**	*b =* 1.56 SU	(−0.06, 3.19)	0.059
Age (55 or older)	26	8,071	*b* = −1.62 SU	(−3.72, 0.48)	0.130	*b =* −1.56 SU	(−3.10, −0.02)	0.047
Education (high)	18	**6,018**	***b* = 2.12 SU**	**(0.18, 4.07)**	**0.033**	*b =* −0.08 SU	(−2.83, 2.68)	0.955
Employment (yes)	16	3,179	*b =* −0.30 SU	(−2.67, 2.07)	0.804	*b =* −0.51 SU	(−2.93, 1.92)	0.682
Partner relationship (yes)	13	6,496	*b =* −0.51 SU	(−2.30, 1.28)	0.576	*b =* −0.45 SU	(−1.92, 1.01)	0.544
***Drinking profiles***								
Regular versus binge-only drinking^3^	27	8,095	*b =* −0.99 SU	(−3.19, 1.21)	0.376	*b =* −0.15 SU	(−1.67, 1.38)	0.851
***Quantity***								
Heavy versus non-heavy drinking^4^	27	8,095	*b =* −1.50 SU	(−3.35, 0.36)	0.114	*b =* −6.03 SU	(−10.23, −1.84)	0.005
***Intervention-level characteristics***								
***Therapeutic guidance***								
Unguided	**19**	**7,366**	***b =* −3.23 SU**	**(−5.88, −0.59)**	**0.017**	*b =* −2.55 SU	(−4.06, −1.04)	0.001
Guided	**8**	**729**	***b =* −10.01 SU**	**(−14.64, −5.39)**	**<0.001**	*b =* −10.68 SU	(−16.80, −4.57)	0.001
Contrast: Guided versus Unguided			***b =* −6.78 SU**	**(−12.11, −1.45)**	**0.013**	*b =* −8.14 SU	(−14.44, −1.84)	0.011
***Intensity***								
Single session	11	3,050	*b =* −3.73 SU	(−7.71, 0.24)	0.066	*b =* −2.97 SU	(−5.14, −0.79)	0.008
Multiple sessions	**16**	**5,045**	***b =* −5.96 SU**	**(−9.31, −2.60)**	**0.001**	*b =* −6.07 SU	(−9.55, −2.60)	0.001
Contrast: Multiple versus Single			*b =* −2.22 SU	(−7.43, 2.98)	0.402	*b =* −3.11 SU	(−7.21, 1.00)	0.138
***Therapeutic orientation***								
Integrated	**18**	**5,550**	***b =* −6.53 SU**	**(−9.57, −3.49)**	**<0.001**	*b =* −6.65 SU	(−9.77, −3.53)	<0.001
PNF only	9	2,545	*b =* −1.98 SU	(−6.24, 2.28)	0.363	*b =* −1.71 SU	(−3.68, 0.27)	0.091
Contrast: PNF versus Integrated			*b =* 4.55 SU	(−0.68, 9.79)	0.088	*b =* 4.94 SU	(1.25, 8.64)	0.009
***Intervention setting***								
Work	3	973	*b =* −1.88 SU	(−9.52, 5.76)	0.630	*b =* −2.69 SU	(−7.61, 2.24)	0.285
Healthcare	**7**	**612**	***b =* −9.12 SU**	**(−14.33, −3.90)**	**0.001**	*b =* −8.08 SU	(−15.88, −0.28)	0.042
Community	**17**	**6,510**	***b =* −4.12 SU**	**(−7.04, −1.19)**	**0.006**	*b =* −3.50 SU	(−5.29, −1.70)	<0.001
***Study-level characteristics***								
***Type of control***								
Waitlist (WLC)	**7**	**647**	***b =* −11.86 SU**	**(−16.01, −7.71)**	**<0.001**	*b =* −11.57 SU	(−17.83, −5.30)	<0.001
Other (AOC or MIC)	**20**	**7,448**	***b =* −2.59 SU**	**(−4.81, −0.38)**	**0.022**	*b =* −2.44 SU	(−3.94, −0.95)	0.001
Contrast: WLC versus Other			***b =* −9.27 SU**	**(−13.97, −4.57)**	**<0.001**	*b =* −9.12 SU	(−15.56, −2.68)	0.005
Assessment only (AOC)	10	2,811	*b =* −0.96 SU	(−4.05, 2.12)	0.541	*b =* −0.88 SU	(−2.16, 0.39)	0.175
Waitlist (WLC)	**7**	**647**	***b =* −11.86 SU**	**(−16.01, −7.71)**	**<0.001**	*b =* −11.57 SU	(−17.83, −5.30)	<0.001
Minimal intervention (MIC)	**10**	**4,637**	***b =* −4.33 SU**	**(−7.51, −1.15)**	**0.008**	*b =* −4.98 SU	(−7.88, −2.07)	0.001
***Interactions***								
Unguided with WLC	**3**	**307**	***b =* −8.25 SU**	**(−14.16, −2.33)**	**0.006**	*b =* −6.59 SU	(−11.57, −1.61)	0.009
Unguided with Other control	16	7,059	*b =* −2.24 SU	(−4.53, 0.07)	0.057	*b =* −2.10 SU	(−3.62, −0.57)	0.007
Contrast: Unguided—WLC versus Other control			*b =* −6.01 SU	(−12.36, 0.34)	0.063	*b =* −4.49 SU	(−9.70, 0.71)	0.091
Guided with WLC	**4**	**340**	***b =* −14.96 SU**	**(−20.47, −9.44)**	**<0.001**	*b =* −14.11 SU	(−23.68, −4.55)	0.004
Guided with Other control	4	389	*b =* −4.45 SU	(−10.06, 1.17)	0.121	*b =* −5.68 SU	(−10.51, −0.84)	0.021
Contrast: Guided—WLC versus Other control			***b =* −10.51 SU**	**(−18.38, −2.64)**	**0.009**	*b =* −8.43 SU	(−19.15, 2.28)	0.123

Significant results are shown in **bold**.

Other control = MIC or AOC.

^1^Studies 36–39 were regarded as outlier studies.

^2^These were studies [52,56].

^3^Regular drinking denotes 14 or more SUs weekly for females or 21 or more for males (thus excluding binge-only drinking). Binge-only drinking denotes more than 4 or 6 SUs (females/males) on at least one occasion per week while still totalling less than 14/21 SUs weekly.

^4^Heavy drinking denotes 35 or more SUs weekly for females and 50 or more for males; non-heavy drinking denotes 14/21 SUs or more, but less than 35/50 SUs, weekly for females/males.

^5^The number of persons refers to respondents for whom data were available on post-intervention drinking behaviour (complete cases) and, for the participant level, also on the moderator in question.

^6^Unstandardised regression coefficients (*b*) indicate the effect of the iAIs in terms of alcohol reduction in SUs.

Abbreviations: AOC, assessment-only control; AUDIT, The Alcohol Use Disorders Identification Test; iAI, internet-based alcohol intervention; MIC, minimal-intervention control (e.g., information brochure); PNF, personalised normative feedback; SU, standard units of alcohol; WLC, waitlist control; 95% CI, 95% confidence interval.

iAI participants also had a significantly greater likelihood of TR than controls (OR = 2.20, 95% CI 1.63–2.95, *p* < 0.001, NNT = 4.15, 95% CI 3.06–6.62), which remained after removal of the outliers (OR = 2.15, 95% CI 1.67–2.77, *p* < 0.001; see Table 4) or the two AUDIT-estimated trials (OR = 2.50, 95% CI 1.81–3.45, *p* < 0.001; see Table 4). Follow-up periods in the analysis were different, but they were not associated with outcomes (primary *p* = 0.41, secondary *p* = 0.12).

**Table 4:** Effects of iAIs in terms of TR (adherence to 14/21 guidelines), moderating effects, and subgroup analyses (one- and two-stage results).

Secondary outcome (TR) and outcome moderators	Comparisons	Persons^4^	One-Stage Results			Two-Stage Results		
			Effect	95% CI	*p*-value	Effect	95% CI	*p*-value
***Overall effect***	**27**	**6,082**	**OR = 2.20**	**(1.63, 2.95)**	**<0.001**	OR = 2.22	(1.58, 3.13)	<0.001
With outlier studies removed^1^	**23**	**2,490**	**OR = 2.15**	**(1.67, 2.77)**	**<0.001**	OR = 2.14	(1.66, 2.76)	<0.001
With studies with AUDIT-only estimates removed^2^	**24**	**5,527**	**OR = 2.50**	**(1.81, 3.45)**	**<0.001**	OR = 2.50	(1.72, 3.63)	<0.001
***Participant-level characteristics***								
***Sociodemographics***								
Gender (female)	27	6,082	OR = 1.00	(0.78, 1.27)	0.982	OR = 0.91	(0.70, 1.18)	0.495
Age (55 or older)	**26**	**6,065**	**OR = 1.66**	**(1.21, 2.27)**	**0.002**	OR = 1.61	(1.15, 2.26)	0.005
Education (high)	18	5,254	OR = 0.92	(0.71, 1.18)	0.493	OR = 1.17	(0.74, 1.86)	0.500
Employment (yes)	16	2,277	OR = 0.86	(0.54, 1.37)	0.535	OR = 0.98	(0.57, 1.68)	0.939
Partner relationship (yes)	14	5,279	OR = 0.96	(0.74, 1.24)	0.743	OR = 0.94	(0.72, 1.23)	0.673
***Quantity***								
Heavy versus non-heavy drinking^3^	27	8,095	OR = 0.94	(0.72, 1.23)	0.646	OR = 0.95	(0.71,1.28)	0.748
***Intervention-level characteristics***								
***Therapeutic guidance***								
Unguided	**19**	**5,544**	**OR = 1.75**	**(1.31, 2.35)**	**<0.001**	OR = 1.72	(1.29, 2.28)	<0.001
Guided	**8**	**538**	**OR = 3.91**	**(2.30, 6.66)**	**<0.001**	OR = 3.97	(1.61, 9.77)	0.003
Contrast: Guided versus Unguided			**OR = 2.23**	**(1.22, 4.08)**	**0.009**	OR = 2.31	(0.90, 5.94)	0.082
***Intensity***								
Single session	**11**	**1,817**	**OR = 1.75**	**(1.14, 2.67)**	**0.010**	OR = 1.66	(1.17, 2.35)	0.004
Multiple sessions	**16**	**4,265**	**OR = 2.58**	**(1.77, 3.75)**	**<0.001**	OR = 2.56	(1.56, 4.21)	<0.001
Contrast: Multiple versus Single			OR = 1.48	(0.84, 2.58)	0.172	OR = 1.54	(0.84, 2.83)	0.160
***Therapeutic orientation***								
Integrated	**18**	**4,819**	**OR = 2.67**	**(1.90, 3.76)**	**<0.001**	OR = 2.77	(1.79, 4.28)	<0.001
PNF only	9	1,263	OR = 1.40	(0.87, 2.25)	0.171	OR = 1.31	(0.93, 1.85)	0.122
Contrast: PNF versus Integrated			**OR = 0.52**	**(0.29, 0.93)**	**0.029**	OR = 0.47	(0.27, 0.83)	0.008
***Intervention setting***								
Work	3	359	OR = 1.23	(0.55, 2.75)	0.610	OR = 1.85	(0.46, 7.46)	0.387
Healthcare	**7**	**435**	**OR = 3.31**	**(1.82, 6.01)**	**<0.001**	OR = 3.08	(0.89, 10.63)	0.074
Community	**17**	**5,288**	**OR = 2.07**	**(1.50, 2.85)**	**<0.001**	OR = 1.95	(1.46, 2.60)	<0.001
***Study-level characteristics***								
***Type of control***								
WLC	**7**	**577**	**OR = 5.79**	**(3.51, 9.55)**	**<0.001**	OR = 5.42	(2.56, 11.46)	<0.001
Other (AOC or MIC)	**20**	**5,505**	**OR = 1.55**	**(1.20, 2.01)**	**0.001**	OR = 1.50	(1.18, 1.91)	0.001
Contrast: WLC versus Other			**OR = 3.74**	**(2.13, 6.53)**	**<0.001**	OR = 3.61	(1.64, 7.93)	0.001
AOC	10	1,529	OR = 1.31	(0.93, 1.86)	0.121	OR = 1.28	(0.96, 1.70)	0.091
WLC	**7**	**577**	**OR = 5.80**	**(3.48, 9.67)**	**<0.001**	OR = 5.42	(2.56, 11.46)	<0.001
MIC	**10**	**3,976**	**OR = 1.87**	**(1.28, 2.75)**	**0.001**	OR = 2.05	(1.28, 3.28)	0.003
***Interactions***								
Unguided with WLC	**3**	**276**	**OR = 3.71**	**(1.86, 7.40)**	**<0.001**	OR = 3.52	(1.90, 6.55)	<0.001
Unguided with Other control	**16**	**5,268**	**OR = 1.48**	**(1.15, 1.90)**	**0.002**	OR = 1.49	(1.15, 1.94)	0.003
Contrast: Unguided—WLC versus Other control			**OR = 2.51**	**(1.21, 5.22)**	**0.014**	OR = 2.36	(1.20, 4.62)	0.012
Guided with WLC	**4**	**301**	**OR = 8.46**	**(4.32, 16.58)**	**<0.001**	OR = 7.62	(2.30, 25.21)	0.001
Guided with Other control	4	237	OR = 1.59	(0.83, 3.06)	0.165	OR = 1.76	(0.75, 4.11)	0.195
Contrast: Guided—WLC versus Other control			**OR = 5.32**	**(2.08, 13.58)**	**<0.001**	OR = 4.34	(1.00, 18.85)	0.050

Significant results are shown in **bold**.

Other control = MIC or AOC.

^1^Studies 36–39 were regarded as outlier studies.

^2^These were studies [52,56].

^3^Heavy drinking denotes 35 or more SUs weekly for females and 50 or more SUs for males; non-heavy drinking denotes 14/21 SUs or more, but less than 35/50 SUs, weekly for females/males.

^4^The number of persons refers to respondents within the subsample of baseline regular drinkers for whom data were available on post-intervention drinking behaviour (complete cases) and, for the participant level, also on the moderator in question. Binge-only drinkers are excluded in this table, as these would have unjustifiably satisfied our mean weekly SU criterion for favourable TR.

Abbreviations: AOC, assessment-only control; AUDIT, The Alcohol Use Disorders Identification Test; iAI, internet-based alcohol intervention; MIC, minimal-intervention control (e.g., information brochure); PNF, personalised normative feedback; SU, standard unit of alcohol consumption based on 10 grams of ethanol; TR, treatment response; WLC, waitlist controlled; 95% CI, 95% confidence interval.

### Moderator analyses

#### Participant characteristics

Both men and women treated in iAIs decreased their mean weekly SU levels to a greater degree than controls, but women did so less than men (2.19 SUs, 95% CI 0.52–3.85, *p* = 0.013). Additional sensitivity analyses maintained this difference. In the first analysis we included only men and women who were exceeding 14 SUs of alcohol at baseline and found a moderator effect of *b* = 2.36 (95% CI 0.41–4.31, *p* = 0.018) for female gender. In the second analysis we included males and females who were exceeding 21 SUs at baseline, resulting in a very similar difference of *b* = 2.52 (95% CI 0.22–4.82, *p* = 0.031). A comparable result emerged for higher- versus lesser-educated participants (2.12 SUs smaller reduction for the former, 95% CI 0.18–4.0, *p* = 0.033). Participants above age 55 were significantly more likely to show TR than younger participants (OR = 1.68, 95% CI 1.22–2.30, *p* = 0.001). No other participant-level moderators were identified for the primary or the secondary outcome.

#### Intervention characteristics

Both unguided (−3.23 SUs, 95% CI −5.88–0.59, *p* = 0.017) and guided iAIs (−10.01 SUs, 95% CI −14.64 to −5.39, *p* < 0.001) were significantly more effective in reducing mean weekly SUs as compared with controls. Guided interventions were significantly more effective than unguided ones (−6.78 SUs, 95% CI −12.11 to −1.45, *p* = 0.013). Similar significant differences in favour of guided iAIs were seen in terms of TR (unguided: OR = 1.75, 95% CI 1.14–2.67, *p* < 0.001; guided: OR = 3.91, 95% CI 2.30–6.66, *p* < 0.001). Guided interventions positively moderated TR likelihood in comparison to unguided ones (OR = 2.23, 95% CI 1.22–4.08, *p* = 0.009). PNF-only interventions showed a significantly lower likelihood of TR than iAIs based on integrated therapeutic principles (OR = 0.52, 95% CI 0.29–0.93, *p* = 0.029). Intervention settings significantly moderated alcohol consumption outcomes for healthcare patients (−9.12 SUs, 95% CI −14.33 to −3.90, *p* = 0.001) and for community populations (−4.12 SUs, 95% CI −7.04 to −1.19, *p* = 0.006) but not for work populations. Similar significant results were evident for TR (healthcare: OR = 3.31, 95% CI 1.82–6.01, *p* < 0.001; community: OR = 2.07, 95% CI 1.50–2.85, *p* < 0.001).

#### Study design characteristics

Treatment participants in waitlist-controlled (WLC) trials significantly reduced their mean weekly alcohol consumption by greater amounts in comparison to controls than those treated in otherwise-controlled trials (−9.27 SUs, 95% CI −13.97 to −4.57, *p* < 0.001). Only the guided iAIs in WLC designs differed significantly from those in otherwise-controlled trials (*b* = −10.51 SUs, 95% CI −18.38 to −2.64, *p* = 0.009). iAI intervention participants in WLC trials were also significantly more likely to show favourable TR (OR = 3.74, 95% CI 2.13–6.53, *p* < 0.001) than those in other trials; significant differences were maintained for both unguided and guided iAIs with WLC designs as compared to other control (unguided: OR = 2.51, 95% CI 1.21–5.22, *p* = 0.014; guided: OR = 5.32, 95% CI 2.08–13.58, *p* < 0.001).

### Sensitivity analyses

In the first sensitivity analysis, we checked the extent to which the results would be different if we used a two-stage approach instead of a one-stage approach. The second sensitivity analysis involved the inclusion of all participants according to the ITT principle by use of a multiple imputation strategy.

The third sensitivity analysis concerned the MAR assumption that is commonly used to deal with missing outcome data. All three of our sensitivity analyses confirmed the results of our main analysis for the overall effect and for most of the moderating effects of participant-, intervention-, and study-level characteristics for the primary and secondary outcomes. This appears to verify the robustness of our findings (see Tables 3 and 4 for the results of the two-stage approach and S2 Table and S3 Table, in which the results of the multiple imputation analyses are presented). Some minimal differences for moderators were seen in the multiple imputation analyses. The moderating role of gender and education for the primary outcome lost significance after multiple imputation. For the secondary outcome, the moderating role of single versus multiple sessions became significantly different in favour of multiple sessions, while intervention in the work setting became effective (*p* = 0.041), as was the case for assessment-only interventions. The contrast between PNF versus integrated iAIs became nonsignificant, as was the case for the contrast between unguided iAIs with WLCs versus other types of control conditions. Thus, in some cases these made our moderator analysis appear more conservative, while in some other cases the MI was more conservative.

**Fig 3:**
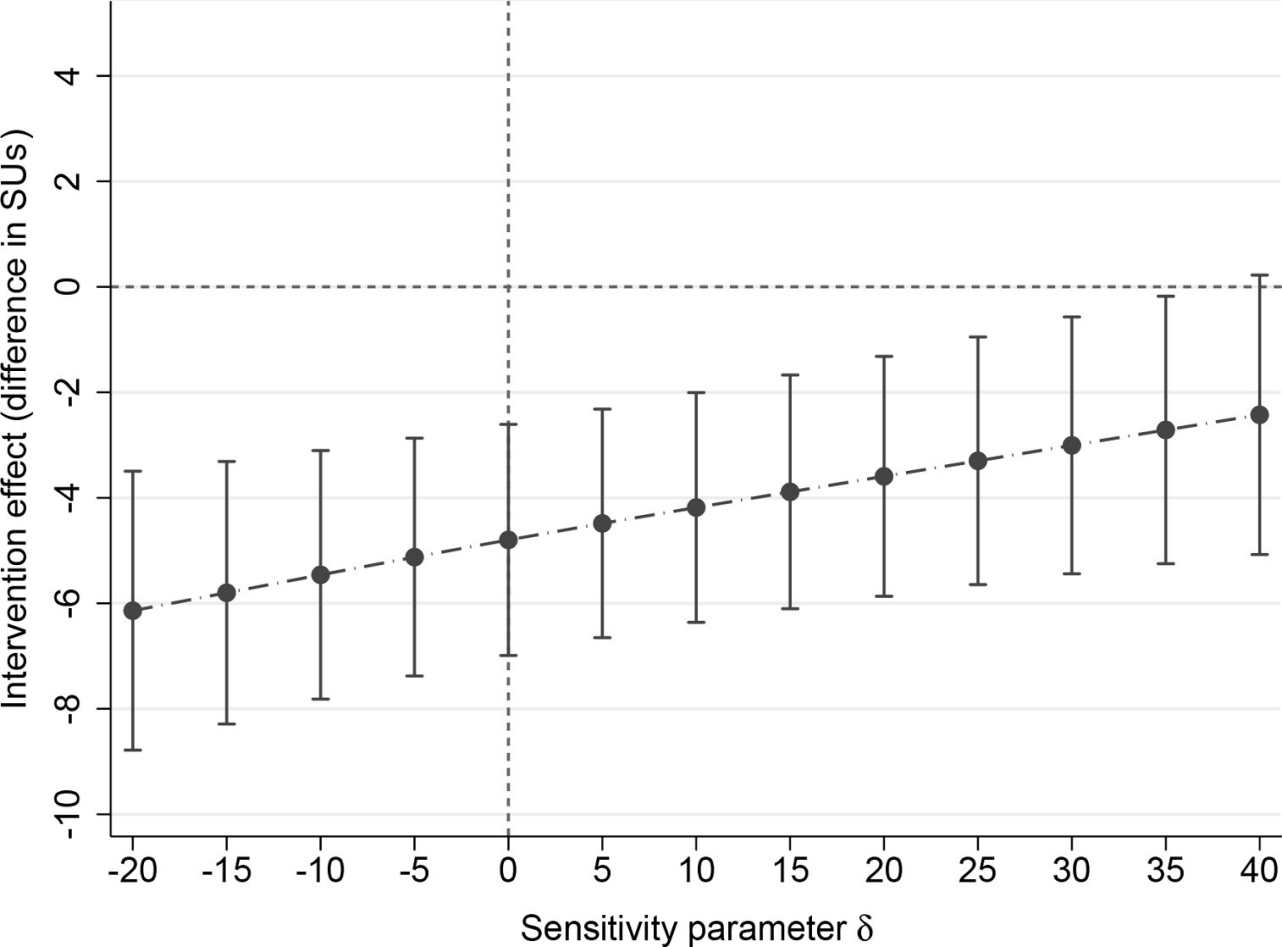
MNAR sensitivity analysis. MNAR, missing-not-at-random; SU, standard unit of alcohol consumption.

Fig 3 depicts the results of the third, MNAR sensitivity analysis, which assessed departure from the MAR assumption. The figure shows estimates (and 95% CIs) of the overall intervention effect on our primary outcome variable, SU, for differing values of δ. The value of δ = 0 corresponds to the MAR assumption, on which the results displayed in Table 3 are based. Positive (or negative) values of δ correspond to situations in which—in each study included in the IPDMA and in both the intervention and the control arms—the mean of unobserved scores for post-intervention SUs would be higher (or lower) than the observed post-intervention SUs, after adjustment for pre-intervention SUs. If MAR holds, the overall effect is estimated in the two-stage method at −4.80 SU. Fig 3 shows that if the post-intervention SUs of dropouts, adjusted for the pre-intervention SUs, were to be 35 SUs higher on average than the post-intervention SUs of participants (being about 1.4 SD above the pre-intervention SUs shown in Table 2), then the estimate of the overall effect would be −4.06 SUs (95% CI −6.25–1.87). If the mean post-intervention SU level of dropouts were to be lower than those of participants (negative value of δ), then the overall effect would be stronger; for instance, if δ = −20, then the estimated overall effect would be −5.32 SUs (95% CI −7.64 to −3.01). This sensitivity analysis leads us to conclude that our results would remain rather stable, even in the event of substantial deviations from the MAR assumption (see S1 Text).

Heterogeneity for the overall primary outcome was high and significant (*I*^*2*^ = 89.6%, CI 78.4%–95.2%, *p* < 0.001) and for the secondary outcome as well (*I*^*2*^ = 78.2%, CI 56.3%–89.9%, *p* < 0.001). It could be partly explained by the identified outliers, as it dropped from high to moderate for the primary outcome (*I*^*2*^ = 55.5%, CI 16.2%–80.3%, *p* < 0.001) and from high to small for the secondary outcome (*I*^*2*^ = 30%, CI 0%–69.1%, *p* < 0.001) after removal of the outliers from the analyses (Tables 3 and 4).

### Conventional meta-analysis comparing included with non-included RCTs

**Fig 4:**
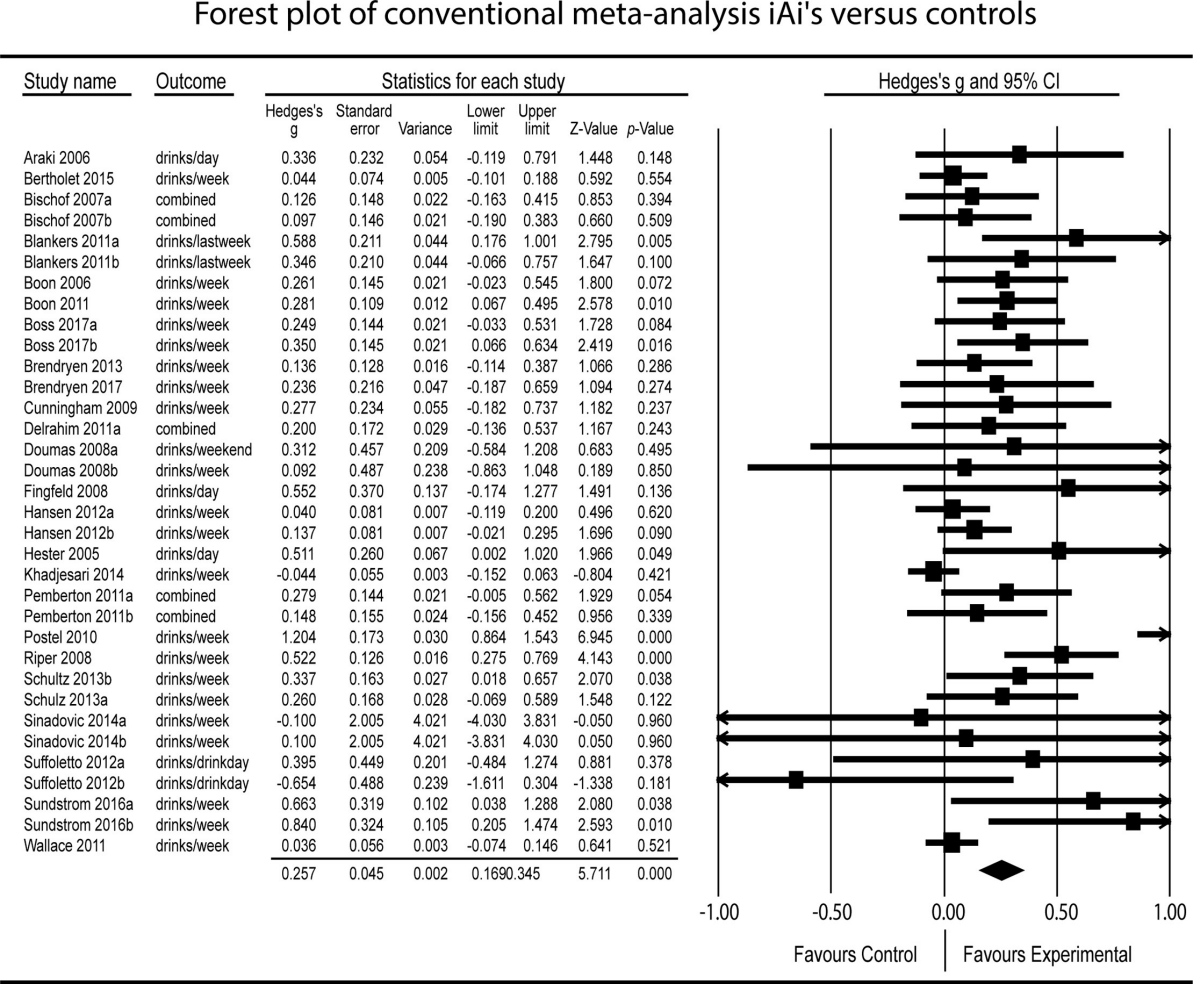
Forest plot of conventional meta-analysis. iAi, internet-based alcohol intervention.

The conventional meta-analysis (24 trials, 34 comparisons) was based on our search up to 31 December 2016 and included additional data from two RCTs published in 2017 [20,47]. It revealed a small significant difference in mean weekly SUs at the first follow-up in favour of iAI participants as compared with controls (Hedges’ *g* = 0.26, 95% CI 0.17–0.34, *p* < 0.001; Fig 4, forest plot of results of conventional meta-analysis). There was significant, moderate heterogeneity, indicating that the effect was greater in some trials than in others (*I*^*2*^ = 65%, *p* < 0.001; 95% CI 49–75). No significant difference in effect size was observed between the included and non-included RCTs in the IPDMA in terms of the primary outcome (SU reduction).

There were indications of publication bias, based on a visual inspection of the funnel plot (see S1 Fig) and Egger test (intercept 1.559, *p* < 0.05), but there was no publication bias observed on the basis of Duval and Tweedie’s trim-and-fill procedure (random-effects model). We could not conduct a conventional meta-analysis for our secondary outcome, as only a limited number of studies reported on it. In S3 Data, this conventional meta-analysis has been expanded with two further eligible studies published between 1 January 2017 and 30 May 2018 that could not be included in our IPDMA. Our aim here was to explore whether more recent studies could potentially alter our IPDMA results; as they did not significantly alter the effect size in our conventional analysis, we believe this confirms the robustness of our analysis.

## Discussion

### Principal findings and their interpretation

This study found that participants treated in iAIs showed a higher mean weekly decrease of 5.02 SUs of alcohol consumption and a greater likelihood of favourable TR (OR 2.20) than controls. Women decreased their mean weekly alcohol consumption significantly less than men (around 2 SUs). Our sensitivity analysis confirmed our assumption that this difference was not an artefact of the higher cutoff thresholds for men than for women at study inclusion (leaving women less space for alcohol reduction) [60]. More highly educated participants reduced their mean weekly consumption significantly less than lesser educated ones (around 2 SUs). This result differs from the few studies that have reported on education as a moderator of iAI treatment outcomes; these showed either improved outcomes for more educated participants [61] or no such impact [62]. For gender and education as moderators of the primary outcome, our sensitivity analyses pointed in similar directions to the outcomes of our main analysis, although the results were no longer significant. In our study, age was found to have moderated TR, with participants above 55 showing greater likelihood of post-intervention adherence to low-risk drinking recommendations than younger people. None of the other participant characteristics moderated treatment outcomes. Internet interventions appear effective when applied in community and healthcare settings, but effectiveness in work settings is still inconclusive.

Guided iAIs yielded significantly better results than unguided ones for both treatment outcomes. iAIs based solely on PNF showed a lower likelihood of TR than iAIs based on integrated therapeutic principles. Waitlist control moderated both types of treatment outcomes, with iAIs in WLC studies showing significantly better outcomes in terms of both SU reduction and TR than those in otherwise-controlled studies. It thus appears that iAI treatment outcomes could have been overestimated in studies in which WLC groups were applied as comparators. One possible explanation for such higher effect sizes in WLC studies would be that problem drinkers allocated to waiting lists might delay their alcohol reduction because they anticipate treatment soon. In contrast, people in other types of control groups might have already found alternative support by the time of the follow-up assessment, thus potentially reducing their alcohol consumption more than WLC controls. By the same token, such tendencies could deflate effect sizes in non-WLC studies [63].

The overall greater reduction of 5.02 SUs of alcohol consumption seen here in iAI treatment participants as compared with controls was higher than the 2.2 SUs we found in our earlier, conventional meta-analysis [14]. One potential explanation for that difference is the higher number of guided iAI studies included in the present IPDMA; these showed higher treatment outcomes than unguided ones. Our current finding is comparable to the 5.61-SU reduction by adult iAI participants over controls reported in the conventional meta-analysis by Kaner and colleagues [16]. Our results compare quite favourably with outcomes of patients treated in primary care settings with brief guided face-to-face interventions, who showed decreases from 2 to 4 SUs [64,65]. We were also able to assess TR in terms of NNT (4.15). Due to data limitations, conventional meta-analyses have not been able to report on NNTs or on potential moderators of iAI treatment such as gender, age, and drinking profiles [16].

### Methodological considerations

To the best of our knowledge, this is the first IPDMA to test the impact of iAIs and their moderators on treatment outcomes with adequate statistical power. The included RCTs had a low overall risk of methodological bias. Our results appear robust after comparison with our two-stage IPDMA results, as well as with those from our multiple imputation analysis and those from our conventional meta-analysis. The ANCOVA model that underlies our IPDMA implicitly relies on the MAR assumption, allowing dropout, which was 43% in our study, to depend on baseline consumption level. Although it cannot be ruled out that dropout was actually attributable to characteristics not included in the model, our MNAR sensitivity analysis suggested that the estimate of the overall effect would be reasonably stable against moderate deviations from the MAR assumption. The generalisability of our results to people in real-life settings might be hampered by poor assessment of ethnicity and by the focus on studies from high-income countries. In addition, only a small number of studies addressed effects of iAIs administered in care settings other than the community (such as in primary care practices, emergency departments, or workplaces). Another limitation is that all studies applied self-reported alcohol consumption measures, which is possibly a source of social desirability bias [66]. We also observed high heterogeneity in our analyses, and it could be explained only partly by excluding outliers or by some of the subgroup analyses that we conducted. Hence, the moderating factors we identified offer only partial clarification of moderating influences on treatment outcome. We were bound, of course, by the available data. Other moderators, such as self-efficacy or participants’ preference for iAIs over other types of interventions, cannot be ruled out [67]. Longer-term outcomes of iAIs could not be assessed, as few studies addressed them.

### Conclusions and clinical implications

Both men and women from different age groups and with different drinking profiles, including heavy drinking and binge-only drinking, can benefit from iAIs, and in particular from the therapeutically integrated ones as opposed to PNF-only interventions. Participants in iAIs reduced their mean alcohol consumption from 38.1 to 32.9 SUs per week, and they had a substantially higher probability of posttreatment adherence to low-risk drinking recommendations. The fact that heavy drinkers decreased their alcohol consumption by amounts similar to those of non-heavy drinkers has favourable implications, as the health impact of a given reduction is greater at higher levels of alcohol consumption [68]. Despite the finding that many participants were still consuming beyond low-risk limits at posttreatment, the population health gains could nevertheless be substantial, in view of the high number of participants that can be reached with iAIs and the positive relationship between decreased alcohol consumption and the lower risks of physical and mental health disorders in the long term. These include earlier-onset dementia [69], several types of cancer [70], cardiovascular diseases [3] (Wood 2018), and depression and anxiety [68,69,71]. iAIs have great scaling potential, partly by virtue of their swift entry procedures for patients and the relatively low cost of repeated reuse, especially if unguided. For many people, iAIs could serve as a first step towards changing their problem-drinking behaviours and towards more intensive treatment, if needed.

In view of the constraints experienced with face-to-face BAIs in primary care settings, future studies should also explore various types of brief interventions, in order to gauge how problem drinkers in such settings can best be targeted. Those could be either face-to-face BAIs or iAIs, and the latter could be guided by general practitioners (GPs) or other professionals. For some patient populations, referral to unguided forms could be more beneficial [72]. More primary care studies are needed, however, including head-to-head comparisons of unguided versus guided versus face-to-face interventions. The same applies to the optimum treatment orientations and levels of intensity and duration [73,74]. As we have seen, not all treatment participants benefited from iAIs. We therefore need to better understand for which people such interventions work, how they work, and in what contexts (an approach also highlighted by Babor in 2008) [75,76]. A final observation is that some countries have now substantially lowered the advised limits for daily and weekly alcohol consumption, in response to mounting epidemiological evidence of health risks inherent in the conventional limits [77]. A threshold not exceeding 10 SUs of weekly alcohol consumption for both men and women has been proposed [3]. Future studies should correspondingly adjust their sample inclusion criteria based on units of alcohol consumption.

## Supporting information

S1 PRISMA guidelines checklist (DOCX)

S1 Data Search string. (DOCX)

S2 Data Request sharing variables file regarding individual RCT results. RCT, randomised controlled trial.
(DOC)

S3 Data Results, conventional meta-analysis update (1 January 2017 and 30 May 2018). (DOCX)

S4 Data Data access contact list (first contact excluding coauthors). (XLSX)

S1 Text MNAR additional information. MNAR, missing-not-at-random.
(DOCX)

S1 Table Risk-of-bias assessment. (DOCX)

S2 Table Results, primary outcome ‘multiple imputation’. (DOCX)

S3 Table Results, secondary outcome ‘multiple imputation’. (DOCX)

S1 Fig Publication bias funnel plot (conventional meta-analysis). (TIF)

## Abbreviations

AUDIT: The Alcohol Use Disorders Identification Test
AUDIT-C: The Alcohol Use Disorders Identification Test (brief, 3 items)
BAI: brief alcohol intervention
BSC: behavioural self-control
CBT: cognitive-behavioural therapy
FAST: Fast Alcohol Screening Test
GP: general practitioner
iAI: internet-based alcohol intervention
IPD: individual patient data
IPDMA: individual patient data meta-analysis
ITT: intention-to-treat
LMM: linear mixed model
MAR: missing at random
MCAR: missing completely at random
MI: motivational interviewing
MNAR: missing-not-at-random
NNT: number needed to treat
OR: odds ratio
PNF: personalised normative feedback
RCT: randomised controlled trial
SU: standard unit of alcohol consumption based on 10 grams of ethanol
TLFB: time line follow-back
TR: treatment response
WLC: waitlist-controlled

## References

[1] Whiteford HA, Degenhardt L, Rehm J, Baxter AJ, Ferrari AJ, Erskine HE, et al Global burden of disease attributable to mental and substance use disorders: findings from the Global Burden of Disease Study 2010. Lancet. 2013;382(9904):1575–86. 10.1016/S0140-6736(13)61611-6 23993280

[2] Kelly S, Olanrewaju O, Cowan A, Brayne C, Lafortune L. Alcohol and older people: A systematic review of barriers, facilitators and context of drinking in older people and implications for intervention design. PLoS ONE. 2018;13(1):e0191189 Epub 2018/01/26. 10.1371/journal.pone.0191189 29370214 PMC5784942

[3] Wood AM, Kaptoge S, Butterworth AS, Willeit P, Warnakula S, Bolton T, et al Risk thresholds for alcohol consumption: combined analysis of individual-participant data for 599 912 current drinkers in 83 prospective studies. Lancet. 2018;391(10129):1513–23. Epub 2018/04/21. 10.1016/S0140-6736(18)30134-X 29676281 PMC5899998

[4] Angus C, Latimer N, Preston L, Li J, Purshouse R. What are the Implications for Policy Makers? A Systematic Review of the Cost-Effectiveness of Screening and Brief Interventions for Alcohol Misuse in Primary Care. Frontiers in psychiatry. 2014;5:114 Epub 2014/09/17. 10.3389/fpsyt.2014.00114 25225487 PMC4150206

[5] Rehm J, Manthey J, Struzzo P, Gual A, Wojnar M. Who receives treatment for alcohol use disorders in the European Union? A cross-sectional representative study in primary and specialized health care. Eur Psychiatry. 2015;30(8):885–93. Epub 2015/12/10. 10.1016/j.eurpsy.2015.07.012 26647862

[6] Excellence NIfHaC. Alcohol-Use Disorders: Diagnosis, Assessment and Management of Harmful Drinking and Alcohol Dependence. Leicester (UK): The British Psychological Society & The Royal College of Psychiatrists; 2011. 22624177

[7] Chan KK, Neighbors C, Gilson M, Larimer ME, Alan Marlatt G. Epidemiological trends in drinking by age and gender: providing normative feedback to adults. Addict Behav. 2007;32(5):967–76. Epub 2006/08/30. 10.1016/j.addbeh.2006.07.003 16938410

[8] Miller WR, Rollnick S. Motivational Interviewing: Preparing people to change addictive behavior. New York: Guilford Press; 2002.

[9] Kalden R, Caroll K, Donovan D, Cooney N, Monti P, Abrams D, et al Cognitive behavioral Coping Skills Therapy Manual: A Clinical Research Guide for Therapists Treating Individuals With Alcohol Abuse and Dependence. In: Mattson MEM, editor. NIAAA Project MATCH Monograph Series. 3 Rockville, Md: National Institute on Alcoholism and Alcohol Abuse; 1992.

[10] Hester RK. Behavioral self-control training In: Hester RK, Miller WR, editors. Handbook of Alcoholism Treatment Approaches: Effective Alternatives. 2nd. Needham Heights, MA: Allyn and Bacon; 1995 p. 148–59.

[11] McCambridge J, Saitz R. Rethinking brief interventions for alcohol in general practice. BMJ. 2017;356:j116 Epub 2017/01/22. 10.1136/bmj.j116 28108452

[12] Riper H, Cuijpers P. Telepsychology and eHealth In: Norcross JCV, G.R.; Freedheim D.K., editor. APA handbook of clinical psychology: Applications and methods (Vol 3). 3: Applications and Methods: American Psychological Association; 2016 p. 451–63.

[13] Wallace P, Bendtsen P. Internet applications for screening and brief interventions for alcohol in primary care settings—implementation and sustainability. Frontiers in psychiatry. 2014;5:151 Epub 2014/11/18. 10.3389/fpsyt.2014.00151 25400593 PMC4214221

[14] Riper H, Blankers M, Hadiwijaya H, Cunningham J, Clarke S, Wiers R, et al Effectiveness of guided and unguided low-intensity internet interventions for adult alcohol misuse: a meta-analysis. PLoS ONE. 2014;9(6):e99912 Epub 2014/06/18. 10.1371/journal.pone.0099912 24937483 PMC4061051

[15] Sijborn M, Luijkx H, Boomsma L, Larsen IM, Burgers J, van der Weele G. [The Dutch College of General Practitioners' practice guideline 'Problem drinking']. Ned Tijdschr Geneeskd. 2015;159:A8646 Epub 2015/03/26. 25804112

[16] Kaner EFS, Beyer FR, Garnett C, Crane D, Brown J, Muirhead C, et al Personalised digital interventions for reducing hazardous and harmful alcohol consumption in community-dwelling populations. Cochrane Database of Systematic Reviews. 2017;9:Cd011479 Epub 2017/09/26. 10.1002/14651858.CD011479.pub2 28944453 PMC6483779

[17] Khadjesari Z, Murray E, Hewitt C, Hartley S, Godfrey C. Can stand-alone computer-based interventions reduce alcohol consumption? A systematic review. Addiction. 2011;106(2):267–82. Epub 2010/11/19. 10.1111/j.1360-0443.2010.03214.x 21083832

[18] Richards D, Richardson T. Computer-based psychological treatments for depression: a systematic review and meta-analysis. Clin Psychol Rev. 2012;32(4):329–42. Epub 2012/04/03. 10.1016/j.cpr.2012.02.004 22466510

[19] Boss L, Lehr D, Berking M, Riper H, Schaub MP, Ebert DD. Evaluating the (cost-) effectiveness of guided and unguided Internet-based self-help for problematic alcohol use in employees—a three arm randomized controlled trial. BMC Public Health. 2015;15:1043 Epub 2015/10/16. 10.1186/s12889-015-2375-0 26458872 PMC4603803

[20] Brendryen H, Johansen A, Duckert F, Nesvag S. A Pilot Randomized Controlled Trial of an Internet-Based Alcohol Intervention in a Workplace Setting. Int J Behav Med. 2017;24(5):768–77. Epub 2017/07/30. 10.1007/s12529-017-9665-0 28755326

[21] Higgins JPT, Green S. Higgins JPT, Green S (editors). Cochrane Handbook for Systematic Reviews of Interventions Version 5.1.0. https://training.cochrane.org/handbook. 2011.

[22] Mongan DJL J. Standard drink measures throughout Europe; peoples’ understanding of standard drinks and their use in drinking guidelines, alcohol surveys and labelling. Dublin: 2015.

[23] Bush K, Kivlahan DR, McDonell MB, Fihn SD, Bradley KA. The AUDIT alcohol consumption questions (AUDIT-C): an effective brief screening test for problem drinking. Ambulatory Care Quality Improvement Project (ACQUIP). Alcohol Use Disorders Identification Test. Arch Intern Med. 1998;158(16):1789–95. Epub 1998/09/17. 9738608 10.1001/archinte.158.16.1789

[24] Khadjesari Z, Freemantle N, Linke S, Hunter R, Murray E. Correction: Health on the web: randomised controlled trial of online screening and brief alcohol intervention delivered in a workplace setting. PLoS ONE. 2015;10(4):e0127371 Epub 2015/04/29. 10.1371/journal.pone.0127371 25915505 PMC4410936

[25] Burke DL, Ensor J, Riley RD. Meta-analysis using individual participant data: one-stage and two-stage approaches, and why they may differ. Stat Med. 2017;36(5):855–75. Epub 2016/10/18. 10.1002/sim.7141 27747915 PMC5297998

[26] Vickers AJ, Altman DG. Statistics notes: Analysing controlled trials with baseline and follow up measurements. BMJ. 2001;323(7321):1123–4. Epub 2001/11/10. 11701584 10.1136/bmj.323.7321.1123 PMC1121605

[27] White IR, Thompson SG. Adjusting for partially missing baseline measurements in randomized trials. Stat Med. 2005;24(7):993–1007. Epub 2004/12/01. 10.1002/sim.1981 15570623

[28] Szumilas M. Explaining odds ratios. J Can Acad Child Adolesc Psychiatry. 2010;19(3):227–9. Epub 2010/09/16. 20842279 PMC2938757

[29] Kraemer HC, Kupfer DJ. Size of treatment effects and their importance to clinical research and practice. BiolPsychiatry. 2006;59(11):990–6. 10.1016/j.biopsych.2005.09.014 16368078

[30] White IR, Horton NJ, Carpenter J, Pocock SJ. Strategy for intention to treat analysis in randomised trials with missing outcome data. BMJ. 2011;342:d40 Epub 2011/02/09. 10.1136/bmj.d40 21300711 PMC3230114

[31] Cohen J. Statistical power analyses for the behavioral sciences (revised ed.). New York: Academic Press; 1997.

[32] Orsini N. BM, Higgins J., Buchan I. HETEROGI: Stata module to quantify heterogeneity in a meta‐analysis. Boston: Boston, MA: Department of Economics, 2006. p. Statistical Software Components.

[33] Ioannidis JP, Patsopoulos NA, Evangelou E. Uncertainty in heterogeneity estimates in meta-analyses. BMJ. 2007;335(7626):914–6. Epub 2007/11/03. 10.1136/bmj.39343.408449.80 17974687 PMC2048840

[34] Stewart LA, Clarke M, Rovers M, Riley RD, Simmonds M, Stewart G, et al Preferred Reporting Items for Systematic Review and Meta-Analyses of individual participant data: the PRISMA-IPD Statement. JAMA. 2015;313(16):1657–65. Epub 2015/04/29. 10.1001/jama.2015.3656 25919529

[35] Delrahim-Howlett K, Chambers CD, Clapp JD, Xu R, Duke K, Moyer RJ 3rd, et al Web-based assessment and brief intervention for alcohol use in women of childbearing potential: a report of the primary findings. Alcohol Clin Exp Res. 2011;35(7):1331–8. Epub 2011/03/18. 10.1111/j.1530-0277.2011.01469.x 21410488

[36] Doumas DM, Hannah E. Preventing high-risk drinking in youth in the workplace: a web-based normative feedback program. J Subst Abuse Treat. 2008;34(3):263–71. Epub 2007/06/30. 10.1016/j.jsat.2007.04.006 17600650

[37] Finfgeld-Connett D. Web-based treatment for rural women with alcohol problems: preliminary findings. Comput Inform Nurs. 2009;27(6):345–53. Epub 2009/11/11. 10.1097/NCN.0b013e3181bca64b 19901570 PMC2819034

[38] Pemberton MR, Williams J, Herman-Stahl M, Calvin SL, Bradshaw MR, Bray RM, et al Evaluation of two web-based alcohol interventions in the U.S. military. Journal of studies on alcohol and drugs. 2011;72(3):480–9. Epub 2011/04/26. 21513685 10.15288/jsad.2011.72.480

[39] Boon B. Preventie problematisch alcoholgebruik In: Meijer SA, Smit F, Schoemaker CG, Cuijpers P, editors. Gezond verstand Evidence-based preventie van psychische stoornissen [Common sense Evidence-based prevention of mental disorders ] VTV Themarapport. Bilthoven: RIVM; 2006.

[40] Saunders JB, Aasland OG, Babor TF, de lF Jr., Grant M. Development of the Alcohol Use Disorders Identification Test (AUDIT): WHO Collaborative Project on Early Detection of Persons with Harmful Alcohol Consumption—II. Addiction. 1993;88(6):791–804. 8329970 10.1111/j.1360-0443.1993.tb02093.x

[41] Hodgson R, Alwyn T, John B, Thom B, Smith A. The FAST Alcohol Screening Test. Alcohol Alcohol. 2002;37(1):61–6. Epub 2002/02/05. 11825859 10.1093/alcalc/37.1.61

[42] Araki I, Hashimoto H, Kono K, Matsuki H, Yano E. Controlled trial of worksite health education through face-to-face counseling vs. e-mail on drinking behavior modification. J Occup Health. 2006;48(4):239–45. Epub 2006/08/12. 16902267 10.1539/joh.48.239

[43] Bertholet N, Cunningham JA, Faouzi M, Gaume J, Gmel G, Burnand B, et al Internet-based brief intervention for young men with unhealthy alcohol use: a randomized controlled trial in a general population sample. Addiction. 2015;110(11):1735–43. Epub 2015/07/16. 10.1111/add.13051 26173842

[44] Bischof G, Grothues JM, Reinhardt S, Meyer C, John U, Rumpf HJ. Evaluation of a telephone-based stepped care intervention for alcohol-related disorders: a randomized controlled trial. Drug Alcohol Depend. 2008;93(3):244–51. 10.1016/j.drugalcdep.2007.10.003 18054443

[45] Blankers M, Koeter MW, Schippers GM. Internet therapy versus internet self-help versus no treatment for problematic alcohol use: A randomized controlled trial. J Consult Clin Psychol. 2011;79(3):330–41. Epub 2011/05/04. 10.1037/a0023498 21534652

[46] Boon B, Risselada A, Huiberts A, Riper H, Smit F. Curbing alcohol use in male adults through computer generated personalized advice: randomized controlled trial. J Med Internet Res. 2011;13(2):e43 Epub 2011/07/02. 10.2196/jmir.1695 21719412 PMC3221373

[47] Boß L, Lehr D, Schaub MP, Paz Castro R, Riper H, Berking M, et al Efficacy of a web-based intervention with and without guidance for employees with risky drinking: results of a three-arm randomized controlled trial. Addiction. 2018;113(4):635–46. Epub 2017/11/07. 10.1111/add.14085 29105879 PMC5887885

[48] Brendryen H, Lund IO, Johansen AB, Riksheim M, Nesvag S, Duckert F. Balance—a pragmatic randomized controlled trial of an online intensive self-help alcohol intervention. Addiction. 2014;109(2):218–26. Epub 2013/10/19. 10.1111/add.12383 24134709

[49] Cunningham JA, Wild TC, Cordingley J, van Mierlo T, Humphreys K. A randomized controlled trial of an internet-based intervention for alcohol abusers. Addiction. 2009;104(12):2023–32. Epub 2009/11/20. 10.1111/j.1360-0443.2009.02726.x 19922569 PMC2779998

[50] Hansen AB, Becker U, Nielsen AS, Gronbaek M, Tolstrup JS, Thygesen LC. Internet-based brief personalized feedback intervention in a non-treatment-seeking population of adult heavy drinkers: a randomized controlled trial. J Med Internet Res. 2012;14(4):e98 Epub 2012/08/01. 10.2196/jmir.1883 22846542 PMC3409578

[51] Hester RK, Squires DD, Delaney HD. The Drinker's Check-up: 12-month outcomes of a controlled clinical trial of a stand-alone software program for problem drinkers. J Subst Abuse Treat. 2005;28(2):159–69. Epub 2005/03/23. 10.1016/j.jsat.2004.12.002 15780546

[52] Khadjesari Z, Freemantle N, Linke S, Hunter R, Murray E. Health on the web: randomised controlled trial of online screening and brief alcohol intervention delivered in a workplace setting. PLoS ONE. 2014;9(11):e112553 Epub 2014/11/20. 10.1371/journal.pone.0112553 25409454 PMC4237335

[53] Postel MG, de Haan HA, ter Huurne ED, Becker ES, de Jong CA. Effectiveness of a web-based intervention for problem drinkers and reasons for dropout: randomized controlled trial. J Med Internet Res. 2010;12(4):e68 Epub 2010/12/18. 10.2196/jmir.1642 21163776 PMC3056532

[54] Riper H, Kramer J, Smit F, Conijn B, Schippers G, Cuijpers P. Web-based self-help for problem drinkers: a pragmatic randomized trial. Addiction. 2008;103(2):218–27. Epub 2008/01/18. 10.1111/j.1360-0443.2007.02063.x 18199300

[55] Schulz DN, Candel MJ, Kremers SP, Reinwand DA, Jander A, de Vries H. Effects of a Web-based tailored intervention to reduce alcohol consumption in adults: randomized controlled trial. J Med Internet Res. 2013;15(9):e206 Epub 2013/09/21. 10.2196/jmir.2568 24045005 PMC3785997

[56] Sinadinovic K, Wennberg P, Johansson M, Berman AH. Targeting individuals with problematic alcohol use via Web-based cognitive-behavioral self-help modules, personalized screening feedback or assessment only: a randomized controlled trial. Eur Addict Res. 2014;20(6):305–18. Epub 2014/10/11. 10.1159/000362406 25300885

[57] Suffoletto B, Kristan J, Callaway C, Kim KH, Chung T, Monti PM, et al A text message alcohol intervention for young adult emergency department patients: a randomized clinical trial. Ann Emerg Med. 2014;64(6):664–72 e4. Epub 2014/07/16. 10.1016/j.annemergmed.2014.06.010 25017822 PMC4254153

[58] Sundstrom C, Gajecki M, Johansson M, Blankers M, Sinadinovic K, Stenlund-Gens E, et al Guided and Unguided Internet-Based Treatment for Problematic Alcohol Use—A Randomized Controlled Pilot Trial. PLoS ONE. 2016;11(7):e0157817 Epub 2016/07/08. 10.1371/journal.pone.0157817 27383389 PMC4934861

[59] Wallace P, Murray E, McCambridge J, Khadjesari Z, White IR, Thompson SG, et al On-line randomized controlled trial of an internet based psychologically enhanced intervention for people with hazardous alcohol consumption. PLoS ONE. 2011;6(3):e14740 Epub 2011/03/17. 10.1371/journal.pone.0014740 21408060 PMC3052303

[60] Cunningham JA. Unintended impact of using different inclusion cut-offs for males and females in intervention trials for hazardous drinking. Addiction. 2017;112(5):910–1. Epub 2017/02/09. 10.1111/add.13760 28168847

[61] Riper H, Kramer J, Keuken M, Smit F, Schippers G, Cuijpers P. Predicting successful treatment outcome of web-based self-help for problem drinkers: secondary analysis from a randomized controlled trial. J Med Internet Res. 2008;10(4):e46 Epub 2008/11/27. 10.2196/jmir.1102 19033150 PMC2629366

[62] Blankers M, Koeter MW, Schippers GM. Baseline predictors of treatment outcome in Internet-based alcohol interventions: a recursive partitioning analysis alongside a randomized trial. BMC Public Health. 2013;13:455 Epub 2013/05/09. 10.1186/1471-2458-13-455 23651767 PMC3662562

[63] Gold SM, Enck P, Hasselmann H, Friede T, Hegerl U, Mohr DC, et al Control conditions for randomised trials of behavioural interventions in psychiatry: a decision framework. The lancet Psychiatry. 2017;4(9):725–32. Epub 2017/04/12. 10.1016/S2215-0366(17)30153-0 28396067

[64] Jonas DE, Garbutt JC, Amick HR, Brown JM, Brownley KA, Council CL, et al Behavioral counseling after screening for alcohol misuse in primary care: a systematic review and meta-analysis for the U.S. Preventive Services Task Force. AnnInternMed. 2012;157(9):645–54. 10.7326/0003-4819-157-9-201211060-00544 23007881

[65] Kaner EF, Beyer FR, Muirhead C, Campbell F, Pienaar ED, Bertholet N, et al Effectiveness of brief alcohol interventions in primary care populations. The Cochrane database of systematic reviews. 2018;2:CD004148. Epub 2018/02/25. 10.1002/14651858.CD004148.pub4 29476653 PMC6491186

[66] Del Boca FK, Darkes J. The validity of self-reports of alcohol consumption: state of the science and challenges for research. Addiction. 2003;98 Suppl 2:1–12. Epub 2004/02/27. 14984237 10.1046/j.1359-6357.2003.00586.x

[67] Babor TF, Caetano R. The trouble with alcohol abuse: what are we trying to measure, diagnose, count and prevent? Addiction. 2008;103(7):1057–9. Epub 2008/06/17. 10.1111/j.1360-0443.2008.02263.x 18554338

[68] Rehm J, Roerecke M. Reduction of drinking in problem drinkers and all-cause mortality. Alcohol Alcohol. 2013;48(4):509–13. Epub 2013/03/28. 10.1093/alcalc/agt021 23531718

[69] Schwarzinger M, Pollock BG, Hasan OSM, Dufouil C, Rehm J, QalyDays Study G. Contribution of alcohol use disorders to the burden of dementia in France 2008–13: a nationwide retrospective cohort study. The Lancet Public health. 2018;3(3):e124–e32. Epub 2018/02/25. 10.1016/S2468-2667(18)30022-7 29475810

[70] Bagnardi V, Rota M, Botteri E, Tramacere I, Islami F, Fedirko V, et al Alcohol consumption and site-specific cancer risk: a comprehensive dose-response meta-analysis. Br J Cancer. 2015;112(3):580–93. Epub 2014/11/26. 10.1038/bjc.2014.579 25422909 PMC4453639

[71] Boschloo L, Vogelzangs N, Smit JH, Van den Brink W, Veltman DJ, Beekman AT, et al Comorbidity and risk indicators for alcohol use disorders among persons with anxiety and/or depressive disorders: findings from the Netherlands Study of Depression and Anxiety (NESDA). JAffectDisord. 2011;131(1–3):233–42. 10.1016/j.jad.2010.12.014 21247636

[72] Wallace P, Struzzo P, Della Vedova R, Scafuri F, Tersar C, Lygidakis C, et al Randomised controlled non-inferiority trial of primary care-based facilitated access to an alcohol reduction website. BMJ open. 2017;7(11):e014576 Epub 2017/11/06. 10.1136/bmjopen-2016-014576 29102982 PMC5722079

[73] Elzerbi C, Donoghue K, Drummond C. A comparison of the efficacy of brief interventions to reduce hazardous and harmful alcohol consumption between European and non-European countries: a systematic review and meta-analysis of randomized controlled trials. Addiction. 2015;110(7):1082–91. Epub 2015/04/29. 10.1111/add.12960 25916993

[74] Moyer VA, Preventive Services Task F. Screening and behavioral counseling interventions in primary care to reduce alcohol misuse: U.S. preventive services task force recommendation statement. Ann Intern Med. 2013;159(3):210–8. Epub 2013/05/24. 10.7326/0003-4819-159-3-201308060-00652 23698791

[75] Babor TF. Treatment for persons with substance use disorders: mediators, moderators, and the need for a new research approach. IntJMethods PsychiatrRes. 2008;17(1 Suppl):S45–S9. 10.1002/mpr.248 PMC6879080 18543362

[76] Gaume J, McCambridge J, Bertholet N, Daeppen JB. Mechanisms of action of brief alcohol interventions remain largely unknown—a narrative review. Frontiers in psychiatry. 2014;5:108 Epub 2014/09/11. 10.3389/fpsyt.2014.00108 25206342 PMC4143721

[77] Broholm K. GL, Gandin C., Ghirini S., Ghiselli A., Jones L., Martire S., Mongan D. MM, Mäkelä P., Rossi L., Sarrazin D., Scafato E., Schumacher J., R. S. Good practice principles for low risk drinking guidelines. Helsinki: National Institute for Health and Welfare (THL), 2016.

