# Supplementary material for: Effectiveness and treatment moderators of internet interventions for adult problem drinking: An individual patient data meta-analysis of 19 randomised controlled trials
Source: PLoS Med. 2018 Dec 18;15(12):e1002714. doi: 10.1371/journal.pmed.1002714 (PMC6298657; doi:10.1371/journal.pmed.1002714)
Supplement: S1 Data — (DOCX) [file pmed.1002714.s002.docx]

**S1 DATA SEARCH STRING**

We combined free terms and MeSH terms indicating Internet interventions (Internet, web, online, computer) with terms indicative of type of treatment (self-help, brief intervention, treatment, unguided, guided, supported); alcohol misuse (alcohol abuse, dependence, problem drinking, hazardous drinking, harmful drinking); and study design (randomised controlled trial, RCT).

**PUBMED Search String**

“Internet”[All Fields] OR “Web”[All Fields] OR “online”[All Fields] OR “computer”[All Fields] OR “mobile”[All Fields] OR “internet”[MeSH Terms]

“self-help”[All Fields] OR “brief intervention”[All Fields] OR “treatment”[All Fields] OR “unguided”[All Fields] OR “guided”[All Fields] OR “supported”[All Fields] OR “low-intensity”[All Fields] OR “Randomized Controlled Trials as Topic”[MeSH Terms] OR “treatment outcome”[MeSH Terms]

“alcohol abuse”[All Fields] OR “dependence”[All Fields] OR “problem drinking”[All Fields] OR “hazardous drinking”[All Fields] OR “harmful drinking”[All Fields] OR “abstinence”[All Fields] OR “alcohol drinking”[MeSH Terms] OR “Alcoholism”[MeSH Terms]
