# Supplementary material for: Effectiveness and treatment moderators of internet interventions for adult problem drinking: An individual patient data meta-analysis of 19 randomised controlled trials
Source: PLoS Med. 2018 Dec 18;15(12):e1002714. doi: 10.1371/journal.pmed.1002714 (PMC6298657; doi:10.1371/journal.pmed.1002714)
Supplement: S2 Data — RCT, randomised controlled trial. (DOC) [file pmed.1002714.s003.doc]

**S2-Data-Variables Request Individual RCTs**

| **Variable** | **Description** |
| --- | --- |
| Patient ID | Anonymised unique patient identifier |
| Missing Data | Value label used for missing |

| **Demographics** | |
| --- | --- |
| Age | Patient age in years |
| Sex | Patient gender |
| Ethnicity | 0=European; 1=indigenous; 2=Asian; 3=African; 4=American |
| Education | 0=no formal education; 1=education up to high school only; 2=high school education; 3=education after high school; 4=post graduate education |
| Employment | 0=employment full time; 1=employment part-time; 2=unemployed/beneficiary |
| Marital status | 0=Married/defacto; 1=single; 2=divorced/widowed; 3 = living together, not married |

| **Psychosocial** | | | |
| --- | --- | --- | --- |
| Social support measure | please provide us the data labels | | |
| Quality of life-QOLI score |  | | |
| Quality of life-EQ5D score |  | | |
| Disability (Sheenan Disability Scale –SDS) score |  | | |
| Alcohol Use (AUDIT) score |  |  | |
|  | |  |  |
| **Intervention details** | | | |
| Group | Randomized group | | |
| Intention to treat analysis or not |  | | |
| Number of modules completed |  | | |
| Dropouts : a) intervention dropout and b) study dropout |  | | |
| Other Adherence or compliance including actual use of the intervention measured by track and change | Data on whether the intervention was received as allocated , measures of use or measures of number of guidance sessions received | | |

| **Clinical Indicators**  **Primary outcomes** | |
| --- | --- |
| **Baseline and follow up** | Continuous and dichotomous scores at baseline on primary alcohol measures(and follow up) |
| AUDIT-C |  |
| Quantity, e.g. Timeline follow back |  |
| Frequency |  |
| Abstinence |  |
| Physiological measures (e.g.: BAC) |  |

| **Clinical indicators**  **Secondary outcomes** | |
| --- | --- |
| **Baseline and follow up** | Continuous scores at baseline on secondary alcohol instrument (and follow up) |
| Alcohol Problem Index |  |
| Alcohol Problem Scale |  |
| MAST (possible alcohol related problems) |  |
| DrInC (consequences) |  |
| In DUC (consequences) |  |
| RAPI (Rutgers Alcohol Problems Index) |  |
| APQ (Alcohol Problem Questionnaire) |  |

| **Clinical Indicators** | |
| --- | --- |
| Help Seeking History | 0=yes, 1=no |
| Co-morbid Anxiety Disorder | 0=yes, 1=no |
| Co-morbid Depressive Disorder | 0=yes, 1=no |
| Co-morbid Mental Health (MH) disorder | 0=yes, 1=no |
| Co-morbid Physical Health (PH) disorder | 0=yes, 1=no |
