# Supplementary material for: Effectiveness and treatment moderators of internet interventions for adult problem drinking: An individual patient data meta-analysis of 19 randomised controlled trials
Source: PLoS Med. 2018 Dec 18;15(12):e1002714. doi: 10.1371/journal.pmed.1002714 (PMC6298657; doi:10.1371/journal.pmed.1002714)
Supplement: S3 Data — (DOCX) [file pmed.1002714.s004.docx]

**S3-Description of original and updated conventional meta-analyses**

Effect sizes were calculated by subtracting the average post-test score of the iAI condition from the average score of the comparator and dividing the result by the pooled standard deviations of the two groups. This effect size is known as Cohen’s *d*. Using data reported in the published papers, we then calculated the effect sizes in terms of Hedges’s *g* (as Cohen’s *d* is subject to small-sample bias). We adjusted *d* by using a scaling factor, which is multiplied by *d* to arrive at Hedges’s bias-corrected effect size *g*. Effect sizes of approximately 0.80 can

be regarded as large, 0.50 as moderate and 0.20 as small.[1] The difference between studies that provided data (*n*=18) and those that did not (*n*=5) was tested in a subgroup analysis. Heterogeneity was examined by calculating *I^2^*, indicating heterogeneity as a percentage (with 25% considered low, 50% moderate and 75% high).[2] The 95% confidence intervals (CI) around I*^2^* were calculated using the non-central chi-squared-based approach in the heterogi module of Stata. [3] We examined publication bias by visually inspecting the funnel plot, by using the trim and fill procedure and by performing Egger’s test of funnel plot asymmetry.[4,5]

**Flowchart of updated search, 1 January 2017 – 30 May 2018**

**Identification**

**Screening**

**Eligibility**

**Included**

692 references identified by literature search:

- PubMed 48
- Embase 147

- PsycINFO 55
- CINAHL 25
- Science Citation Index Expanded 196
- Social Sciences Citation Index 221
- Arts and Humanities Citation Index 0

After removal of duplicates:

418 abstracts

(274 duplicates removed)

22 publications retrieved

Excluded: 18

- student populations 8

- non-RCT 4

- other type of intervention 6

4 individual randomised trials

(of which 2 were already included before publication via our co-authors)

**Conventional meta-analysis up to 30 May 2018**

In the updated conventional meta-analysis, we found two additional RCT studies that were eligible for inclusion, both evaluating single-session unguided PNF interventions.[6,7] The Guillemont (2017) study was conducted among adult problem drinkers in the community in France, while the Pedersen (2017) study was conducted among US veterans. The updated analysis (26 studies, 36 comparisons) revealed a small, significant difference in mean weekly SUs at first follow-up in favour of iAI participants as compared to controls (Hedges’s *g* = 0.25, 95% CI 0.17 to 0.33, *p*<0 .001). Those results were almost identical to the effect size we found in our original conventional meta-analysis up to 31 December 2016, which revealed a Hedges’s *g* of 0.26 (95% CI 0.17 to 0.34, *p*<0.001).

**References**

1. Cohen J. Statistical power analyses for the behavioral sciences (revised ed.). New York: Academic Press; 1997.

2. Higgins JP, Thompson SG, Deeks JJ, Altman DG. Measuring inconsistency in meta-analyses. BMJ. 2003;327(7414):557-60. Epub 2003/09/06. doi: 10.1136/bmj.327.7414.557. PubMed PMID: 12958120; PubMed Central PMCID: PMCPMC192859.

3. Orsini N. BM, Higgins J., Buchan I. . HETEROGI: Stata module to quantify heterogeneity in a meta‐analysis. Boston: Boston, MA: Department of Economics, ; 2006. p. Statistical Software Components.

4. Duval S, Tweedie R. Trim and fill: A simple funnel-plot-based method of testing and adjusting for publication bias in meta-analysis. Biometrics. 2000;56(2):455-63. Epub 2000/07/06. PubMed PMID: 10877304.

5. Egger M, Davey Smith G, Schneider M, Minder C. Bias in meta-analysis detected by a simple, graphical test. BMJ. 1997;315(7109):629-34. Epub 1997/10/06. PubMed PMID: 9310563; PubMed Central PMCID: PMCPMC2127453.

6. Guillemont J, Cogordan C, Nalpas B, Nguyen-Thanh V, Richard JB, Arwidson P. Effectiveness of a web-based intervention to reduce alcohol consumption among French hazardous drinkers: a randomized controlled trial. Health Educ Res. 2017;32(4):332-42. Epub 2017/09/01. doi: 10.1093/her/cyx052. PubMed PMID: 28854571.

7. Pedersen ER, Parast L, Marshall GN, Schell TL, Neighbors C. A randomized controlled trial of a web-based, personalized normative feedback alcohol intervention for young-adult veterans. J Consult Clin Psychol. 2017;85(5):459-70. Epub 2017/03/14. doi: 10.1037/ccp0000187. PubMed PMID: 28287799; PubMed Central PMCID: PMCPMC5398915.
