# Supplementary material for: Effectiveness and treatment moderators of internet interventions for adult problem drinking: An individual patient data meta-analysis of 19 randomised controlled trials
Source: PLoS Med. 2018 Dec 18;15(12):e1002714. doi: 10.1371/journal.pmed.1002714 (PMC6298657; doi:10.1371/journal.pmed.1002714)
Supplement: S1 Text — MNAR, missing-not-at-random. (DOCX) [file pmed.1002714.s006.docx]

**S1-Text. Sensitivity analysis regarding the MAR assumption**

*Introduction*

The sensitivity analysis to test the missing-at-random (MAR) assumption in our mechanism for missing data caused by study and intervention dropout was performed using Ian White’s *rctmiss* package for Stata.[1-3] The package enables researchers to ‘analyse a randomised controlled trial (RCT) allowing for informatively missing outcome data’. The results of our study, as displayed in tables 4 and 5 (main text), were obtained under the MAR assumption for all studies within our IPDMA. We used *rctmiss* ‘to assess the sensitivity of the results to plausible departures from MAR’.

*The focus of the sensitivity analysis*

The sensitivity analysis was focused on the overall effect of the intervention on the primary outcome, as obtained from the two-stage IPDMA, in which the overall effect was estimated to be −4.80 SUs (a reduction of alcohol consumption), with a 95% confidence interval of −6.99 to −2.61 (see table 4). In the main text of our article, we interpret the results of the sensitivity analysis as follows: ‘Figure 3 shows that if the post-intervention SUs of dropouts, adjusted for the pre-intervention SUs, were to be 35 SUs higher on average than the post-intervention SUs of participants (being about 1.4 standard deviation above the pre-intervention SUs shown in table 2), then the estimate of the overall effect would be −4.06 SUs (95% CI −6.25 to 1.87). If the mean post-intervention SU level of dropouts were to be lower than those of participants (negative value of δ), then the overall effect would be stronger; for instance, if δ=−20, then the estimated overall effect would be −5.32 SUs (95% CI −7.64 to −3.01). This sensitivity analysis leads us to conclude that our results would remain rather stable even in the event of substantial deviations from the MAR assumption.’

*Estimating effects under the MAR assumption*

In a first step of the two-stage IPDMA, ANCOVA models were estimated at the study level, providing estimates and standard errors of the intervention effect from each study under the MAR assumption. In a second step, the estimates and standard errors were combined into an overall estimate and standard error of the intervention effect, using a random effects model and using the restricted maximum likelihood estimation (REML) method. In reality, we used David Fisher’s *ipdmetan* package, which performs the two steps using a single command.[4]

*Estimating effects under departure from MAR*

Estimating the overall effect of the intervention under departure from the MAR assumption also required two steps. In this case, the first step involved obtaining estimates and standard errors of the intervention effect from each study under departure from the MAR assumption, using sensitivity parameter δ. Positive or negative values of δ indicated that respondents who dropped out would have had hypothetically higher (positive δ) or lower (negative δ) values for post-intervention SUs of alcohol than would be expected from the pre-intervention SUs reported by them. Thus, the parameter δ reflects the difference between the means of the observed and the unobserved values of post-intervention SUs, after adjustment for pre-intervention SU levels. We assumed the parameter δ to apply for both the intervention and the control arms of the trial. Having obtained adapted estimates and standard errors that corresponded to a particular value of δ for all studies, we combined the estimates and standard errors in the second step into an overall estimate and standard error of the intervention effect, using a random effects model and using the restricted maximum likelihood estimation (REML) method. For the second step, we used the *admetan* command that comes with the *ipdmetan* package. This process was repeated for several values of δ, including the value of δ=0 (corresponding to the MAR assumption), for which the results were defined as identical to those in table 4.

*Results*

Figure 3 shows results for a series of values of δ:

δ=−2 down to −20: These values of δ reflect situations where dropouts would exhibit *lower* mean post-intervention SU levels than participants, after adjustment for pre-intervention SUs, with a difference of δ SUs, and with δ=0 reflecting MAR.

δ=+2 up to +40: These values of δ reflect situations where dropouts would exhibit *higher* mean post-intervention SU levels than participants, after adjustment for pre-intervention SUs, with a difference of δ SUs, and with δ=0 reflecting MAR.

**References**

1. White IR, Carpenter J, Horton NJ. A mean score method for sensitivity analysis to departures from the missing at random assumption in randomised trials. Statistica Sinica. 2018;28(4):1985-2003. Epub 2018/10/05. doi: 10.5705/ss.202016.0308. PMID: 30283213

2. White IR, Horton NJ, Carpenter J, Pocock SJ. Strategy for intention to treat analysis in randomised trials with missing outcome data. BMJ. 2011;342:d40. Epub 2011/02/09. doi: 10.1136/bmj.d40. PMID: 21300711

3. White IR, Thompson SG. Adjusting for partially missing baseline measurements in randomized trials. Stat Med. 2005;24(7):993-1007. Epub 2004/12/01. doi: 10.1002/sim.1981. PMID: 15570623

4. Fisher DJ. Two-stage individual participant data meta-analysis and generalized forest plots. Stata Journal. 2015;15(2):369-97.
