# Supplementary material for: Effectiveness and treatment moderators of internet interventions for adult problem drinking: An individual patient data meta-analysis of 19 randomised controlled trials
Source: PLoS Med. 2018 Dec 18;15(12):e1002714. doi: 10.1371/journal.pmed.1002714 (PMC6298657; doi:10.1371/journal.pmed.1002714)
Supplement: S1 Table — (DOCX) [file pmed.1002714.s007.docx]

| **S1 Table: Risk-of-bias assessment for included studies (*N* = 19)** | | | | | |
| --- | --- | --- | --- | --- | --- |
|  | **Risk of bias** | | | | |
| **Study** | **Random sequence generation** | **Allocation concealment** | **Blinding of participants**  **and staff** | **Blinding of outcome assessors** | **Incomplete outcome data** |
| Araki et al. 2006 | Unclear | Unclear | High | Low | Low |
| Bertholet et al. 2015 | Low | Low | High | Low | Low |
| Bischof et al. 2008 | Low | Low | High | Low | Low |
| Blankers et al. 2011 | Low | Low | High | Low | Low |
| Boon et al. 2011 | Low | Low | High | Low | Low |
| Brendryen et al. 2014 | Low | Unclear | High | Low | High |
| Brendryen et al. 2017 | Low | Unclear | High | Low | High |
| Cunningham et al.009 | Low | Unclear | High | Low | Low |
| Boß et al. 2017 | Low | Low | High | Low | Low |
| Hansen et al. 2012 | Low | Unclear | High | Low | High |
| Hester et al. 2005 | Unclear | Unclear | High | Low | Low |
| Khadjesari et al. 2014 | Low | Low | High | Low | Low |
| Postel et al. 2014 | Low | Low | High | Low | High |
| Riper et al. 2008 | Low | Low | High | Low | High |
| Schulz et al. 2014 | Low | Low | High | Low | Unclear |
| Sinadinovic et al. 2014 | Low | Low | High | Low | High |
| Suffoletto et al. 2012 | Low | Low | High | Low | Low |
| Sundström et al. 2016 | Low | Low | High | Low | Low |
| Wallace et al. 2011 | Low | Low | Low | Low | High |
