# Supplementary material for: Effectiveness and treatment moderators of internet interventions for adult problem drinking: An individual patient data meta-analysis of 19 randomised controlled trials
Source: PLoS Med. 2018 Dec 18;15(12):e1002714. doi: 10.1371/journal.pmed.1002714 (PMC6298657; doi:10.1371/journal.pmed.1002714)
Supplement: S2 Table — (DOCX) [file pmed.1002714.s008.docx]

**S2 Table: Effects of iAIs in terms of weekly standard units of alcohol (SUs), moderating effects and subgroup analyses. Results of one-stage sensitivity analysis based on multiple imputation.**

|  |  |  | ONE-STAGE RESULTS | | |
| --- | --- | --- | --- | --- | --- |
| **Primary outcome variable and moderators** | Comparisons | Persons | Effect ^4)^ | 95% CI | *P*-value |
|  |  |  |  |  |  |
| ***Overall effect*** | 27 | 14198 | *b*=−5.01 SU | (−7.71, **−**2.30) | **<.001** |
| With outlier studies removed ^1)^ | 23 | 4558 | *b*=−4.89 SU | (−7.16, −2.62) | **<.001** |
| With studies with AUDIT-only estimates removed | 24 | 12455 | *b*=−5.76 SU | (−8.74, −2.78) | **<.001** |
|  |  |  |  |  |  |
| ***Participant-level moderators*** |  |  |  |  |  |
| ***Sociodemographics*** |  |  |  |  |  |
| Gender (female) | 27 | 14198 | *b*=1.15 SU | (−0.46, 2.76) | .160 |
| Age (55 or older) | 27 | 14198 | *b*=−1.14 SU | (−3.31, 1.04) | .305 |
| Education (high) | 27 | 14198 | *b*=0.85 SU | (−0.77, 2.46) | .303 |
| Employment (yes) | 27 | 14198 | *b*=−0.40 SU | (−3.50, 2.69) | .797 |
| Partner relationship (yes) | 27 | 14198 | *b*=−0.26 SU | (−1.91, 1.38) | .755 |
|  |  |  |  |  |  |
| ***Drinking profiles*** |  |  |  |  |  |
| Regular versus binge-only drinking ^2)^ | 27 | 14198 | *b*=−0.66 SU | (−2.76, 1.45) | .541 |
|  |  |  |  |  |  |
| ***Quantity*** |  |  |  |  |  |
| Heavy versus non-heavy drinking ^3)^ | 27 | 14198 | *b*=−0.71 SU | (−2.71, 1.28) | .482 |
|  |  |  |  |  |  |
| ***Intervention characteristics*** |  |  |  |  |  |
| ***Therapeutic guidance*** |  |  |  |  |  |
| Unguided | 19 | 13264 | *b*=−3.09 SU | (−5.86, −0.32) | **.029** |
| Guided | 8 | 934 | *b*=−10.17 SU | (−15.04, −5.31) | **<.001** |
| Contrast: Guided versus Unguided |  |  | *b*=−7.08 SU | (−12.68, −1.49) | **.013** |
|  |  |  |  |  |  |
| ***Intensity*** |  |  |  |  |  |
| Single session | 11 | 4046 | *b*=−3.56 SU | (−7.77, 0.65) | .098 |
| Multiple sessions | 16 | 10152 | *b*=−5.99 SU | (−9.52, −2.46) | .**001** |
| Contrast: Multiple versus Single |  |  | *b*=−2.43 SU | (−7.92, 3.06) | .385 |
|  |  |  |  |  |  |
| ***Therapeutic orientation*** |  |  |  |  |  |
| Integrated | 18 | 10906 | *b*=−6.56 SU | (−9.77, −3.34) | **<.001** |
| PNF only | 9 | 3292 | *b*=−1.72 SU | (−6.23, 2.79) | .455 |
| Contrast: PNF versus Integrated |  |  | *b*=4.84 SU | (−0.70, 10.37) | .087 |
|  |  |  |  |  |  |
| ***Intervention setting*** |  |  |  |  |  |
| Work | 3 | 1259 | *b*=−1.70 SU | (−9.79, 6.38) | .680 |
| Health care | 7 | 765 | *b*=−9.08 SU | (−14.64, −3.51) | **.001** |
| Community | 17 | 12174 | *b*=−4.13 SU | (−7.24, −1.03) | **.009** |
|  |  |  |  |  |  |
| ***Study-level characteristics***  ***Type of control*** |  |  |  |  |  |
| Waitlist (WLC)) | 7 | 861 | *b*=−11.98 SU | (−16.57, −7.39) | **<.001** |
| Other (AOC or MIC) | 20 | 13337 | *b*=−2.65 SU | (−5.10, −0.19) | **.035** |
| Contrast: WLC versus Other |  |  | *b*=−9.34 SU | (−14.53, −4.14) | **<.001** |
|  |  |  |  |  |  |
| Assessment only (AOC) | 10 | 4037 | *b*=−0.78 SU | (−4.09, 2.54) | .646 |
| Waitlist (WLC) | 7 | 861 | *b*=−11.98 SU | (−16.52, −7.44) | **<.001** |
| Minimal intervention (MIC) | 10 | 9300 | *b*=−4.59 SU | (−8.09, −1.09) | **.010** |
|  |  |  |  |  |  |
| ***Interaction*** |  |  |  |  |  |
| Unguided with WLC | 3 | 374 | *b*=−8.06 SU | (−14.67, −1.45) | .**017** |
| Unguided with Other control | 16 | 12890 | *b*=−2.19 SU | (−4.72, 0.34) | .089 |
| Contrast: Unguided – WLC versus Other control |  |  | *b*=−5.87 SU | (−12.94, 1.21) | .104 |
|  |  |  |  |  |  |
| Guided with WLC | 4 | 487 | *b*=−15.09 SU | (−21.09, −9.09) | **<.001** |
| Guided with Other control | 4 | 447 | *b*=−4.93 SU | (−11.15, 1.28) | .120 |
| Contrast: Guided – WLC versus Other control |  |  | *b*=−10.16 SU | (−18.83, −1.49) | .**022** |

Significant results are shown in **bold**. SU: standard units of alcohol; WLC: waitlist control; MIC: minimal-intervention control (e.g. information brochure); AOC: assessment-only control; Other control: MIC or AOC. ^1)^ Studies [1-4] were regarded as outlier studies. ^2)^ Regular drinking denotes 14 or more SUs weekly for females or 21 or more for males (thus excluding binge-only drinking). Binge-only drinking denotes more than 4 or 6 SUs (females/males) on at least one occasion per week, while still totalling less than 14/21 SUs weekly. ^3)^ Heavy drinking denotes 35 or more SUs weekly for females and 50 or more for males; non-heavy drinking denotes 14/21 SUs or more, but less than 35/50 SUs, weekly for females/males. ^4)^ Unstandardised regression coefficients (*b*) indicate the effect of the iAIs in terms of alcohol reduction in SUs.

**Table 5a. Effects of iAIs in terms of treatment response (adherence to 14/21 guidelines), moderating effects and subgroup analyses. Results of one-stage sensitivity analysis based on multiple imputation.**

|  |  |  | ONE-STAGE RESULTS | | |
| --- | --- | --- | --- | --- | --- |
| **Secondary outcome variable and moderators** | Comparisons | Persons ^3) 4)^ | Effect | 95% CI | *P*-value |
|  |  |  |  |  |  |
| ***Overall effect*** | 27 | 13362 | OR=2.05 | (1.52, 2.77) | **<0.001** |
| With outlier studies removed ^1)^ | 23 | 3649 | OR=2.05 | (1.59, 2.66) | **<0.001** |
| With studies with AUDIT-only estimates removed | 24 | 10550 | OR=2.34 | (1.70, 3.22) | **<0.001** |
|  |  |  |  |  |  |
| ***Participant-level moderators*** |  |  |  |  |  |
| ***Sociodemographics*** |  |  |  |  |  |
| Gender (female) | 27 | 13362 | OR=0.94 | (0.77, 1.14) | 0.521 |
| Age (55 or older) | 27 | 13362 | OR=1.38 | (1.04, 1.83) | **0.025** |
| Education (high) | 27 | 13362 | OR=1.11 | (0.91, 1.36) | 0.300 |
| Employment (yes) | 27 | 13362 | OR=1.00 | (0.76, 1.33) | 0.980 |
| Partner relationship (yes) | 27 | 13362 | OR=1.12 | (0.92, 1.37) | 0.252 |
|  |  |  |  |  |  |
| ***Quantity*** |  |  |  |  |  |
| Heavy versus non-heavy drinking ^2)^ | 27 | 13362 | OR=1.17 | (0.93, 1.48) | 0.170 |
|  |  |  |  |  |  |
| ***Intervention characteristics*** |  |  |  |  |  |
| ***Therapeutic guidance*** |  |  |  |  |  |
| Unguided | 19 | 10643 | OR=1.75 | (1.34, 2.29) | **<0.001** |
| Guided | 8 | 720 | OR=3.80 | (2.24, 6.43) | **<0.001** |
| Contrast: Guided versus Unguided |  |  | OR=2.16 | (1.20, 3.90) | **0.010** |
|  |  |  |  |  |  |
| ***Intensity*** |  |  |  |  |  |
| Single session | 11 | 2534 | OR=1.68 | (1.14, 2.49) | **0.009** |
| Multiple sessions | 16 | 8828 | OR=2.52 | (1.83, 3.48) | **<0.001** |
| Contrast: Multiple versus Single |  |  | OR=1.50 | (0.91, 2.47) | **0.115** |
|  |  |  |  |  |  |
| ***Therapeutic orientation*** |  |  |  |  |  |
| Integrated | 18 | 9640 | OR=2.27 | (1.68, 3.07) | **<0.001** |
| PNF only | 9 | 1721 | OR=1.38 | (0.86, 2.21) | 0.184 |
| Contrast: PNF versus Integrated |  |  | OR=0.61 | (0.35, 1.06) | 0.078 |
|  |  |  |  |  |  |
| ***Intervention setting*** |  |  |  |  |  |
| Work | 3 | 468 | OR=2.15 | (1.03, 4.47) | **0.041** |
| Health care | 7 | 571 | OR=3.26 | (1.81, 5.87) | **<0.001** |
| Community | 17 | 10323 | OR=2.16 | (1.61, 2.90) | **<0.001** |
|  |  |  |  |  |  |
| ***Study-level characteristics***  ***Type of control*** |  |  |  |  |  |
| Waitlist (WLC)) | 7 | 768 | OR=5.32 | (2.98, 9.51) | **<0.001** |
| Other (AOC or MIC) | 20 | 10593 | OR=1.98 | (1.48, 2.64) | **<0.001** |
| Contrast: WLC versus Other |  |  | OR=2.69 | (1.41, 5.12) | **0.003** |
|  |  |  |  |  |  |
| Assessment only (AOC) | 10 | 2395 | OR=1.72 | (1.15, 2.57) | **0.009** |
| Waitlist (WLC) | 7 | 769 | OR=5.21 | (2.93, 9.27) | **<0.001** |
| Minimal intervention (MIC) | 10 | 8199 | OR=2.31 | (1.55, 3.47) | **<0.001** |
|  |  |  |  |  |  |
|  |  |  |  |  |  |
| ***Interaction*** |  |  |  |  |  |
| Unguided with WLC | 3 | 334 | OR=3.66 | (1.61, 8.29) | **0.002** |
| Unguided with Other control | 16 | 10308 | OR=1.76 | (1.32, 2.35) | **<0.001** |
| Contrast: Unguided – WLC versus Other control |  |  | OR=2.08 | (0.88, 4.92) | 0.095 |
|  |  |  |  |  |  |
| Guided with WLC | 4 | 434 | OR=7.20 | (3.43, 15.13) | <**0.001** |
| Guided with Other control | 4 | 285 | OR=1.72 | (0.78, 3.81) | 0.182 |
| Contrast: Guided – WLC versus Other control |  |  | OR=4.19 | (1.41, 12.44) | **0.010** |
|  |  |  |  |  |  |
|  |  |  |  |  |  |
|  |  |  |  |  |  |

Significant results are shown in **bold**. 95% CI: 95% confidence interval; WLC: waitlist control; MIC: minimal-intervention control (e.g. information brochure); AOC: assessment-only control; Other control: MIC or AOC. ^1)^ The studies [1-4] were regarded as outlier studies. ^2)^ Heavy drinking denotes 35 or more standard units of alcohol (SUs) weekly for females and 50 or more for males; non-heavy drinking denotes 14/21 SUs or more, but less than 35/50 SUs, weekly for females/males. ^3)^ The number of persons refers to baseline regular drinkers (14 or more SUs weekly for females or 21 or more for males). Non-regular, binge-only drinkers are excluded in this table, as these might have unjustifiably satisfied our mean weekly SU criterion for favourable treatment response. ^4^)The numbers of respondents with drinking behaviours exceeding the guidelines at baseline in fact varied amongst imputation datasets due to selection of target respondents based on imputed baseline-intervention alcohol intake which were missing for a few respondents. As a result, a respondent for which baseline drinking outcome was missing could have been included in one imputation set but not in the other (we imputed 100 times). For example, for the overall effect and the participant-level moderators, the sample size varied slightly between 13357 and 13368, with mean of 13362.5). The subsample sizes shown in the table are rounded upwards.

References

1. Bertholet N, Cunningham JA, Faouzi M, Gaume J, Gmel G, Burnand B, et al. Internet-Based Brief Intervention to Prevent Unhealthy Alcohol Use among Young Men: A Randomized Controlled Trial. PLoS One. 2015;10(12): e0144146. Epub 2015/12/08. doi: 10.1371/journal.pone.0144146. PubMed PMID: 26642329; PubMed Central PMCID: PMCPMC4671673.

2. Khadjesari Z, Freemantle N, Linke S, Hunter R, Murray E. Health on the web: randomised controlled trial of online screening and brief alcohol intervention delivered in a workplace setting. PLoS One. 2014;9(11):e112553. Epub 2014/11/20. doi: 10.1371/journal.pone.0112553. PubMed PMID: 25409454; PubMed Central PMCID: PMCPMC4237335.

3. Postel MG, de Haan HA, ter Huurne ED, Becker ES, de Jong CA. Effectiveness of a web-based intervention for problem drinkers and reasons for dropout: randomized controlled trial. J Med Internet Res. 2010;12(4):e68. Epub 2010/12/18. doi: 10.2196/jmir.1642. PubMed PMID: 21163776; PubMed Central PMCID: PMCPMC3056532.

4. Wallace P, Murray E, McCambridge J, Khadjesari Z, White IR, Thompson SG, et al. On-line randomized controlled trial of an internet based psychologically enhanced intervention for people with hazardous alcohol consumption. PLoS One. 2011;6(3):e14740. Epub 2011/03/17. doi: 10.1371/journal.pone.0014740. PubMed PMID: 21408060; PubMed Central PMCID: PMCPMC3052303.
