# Supplementary figures and images for: Effectiveness and treatment moderators of internet interventions for adult problem drinking: An individual patient data meta-analysis of 19 randomised controlled trials
Source: PLoS Med. 2018 Dec 18;15(12):e1002714. doi: 10.1371/journal.pmed.1002714 (PMC6298657; doi:10.1371/journal.pmed.1002714)

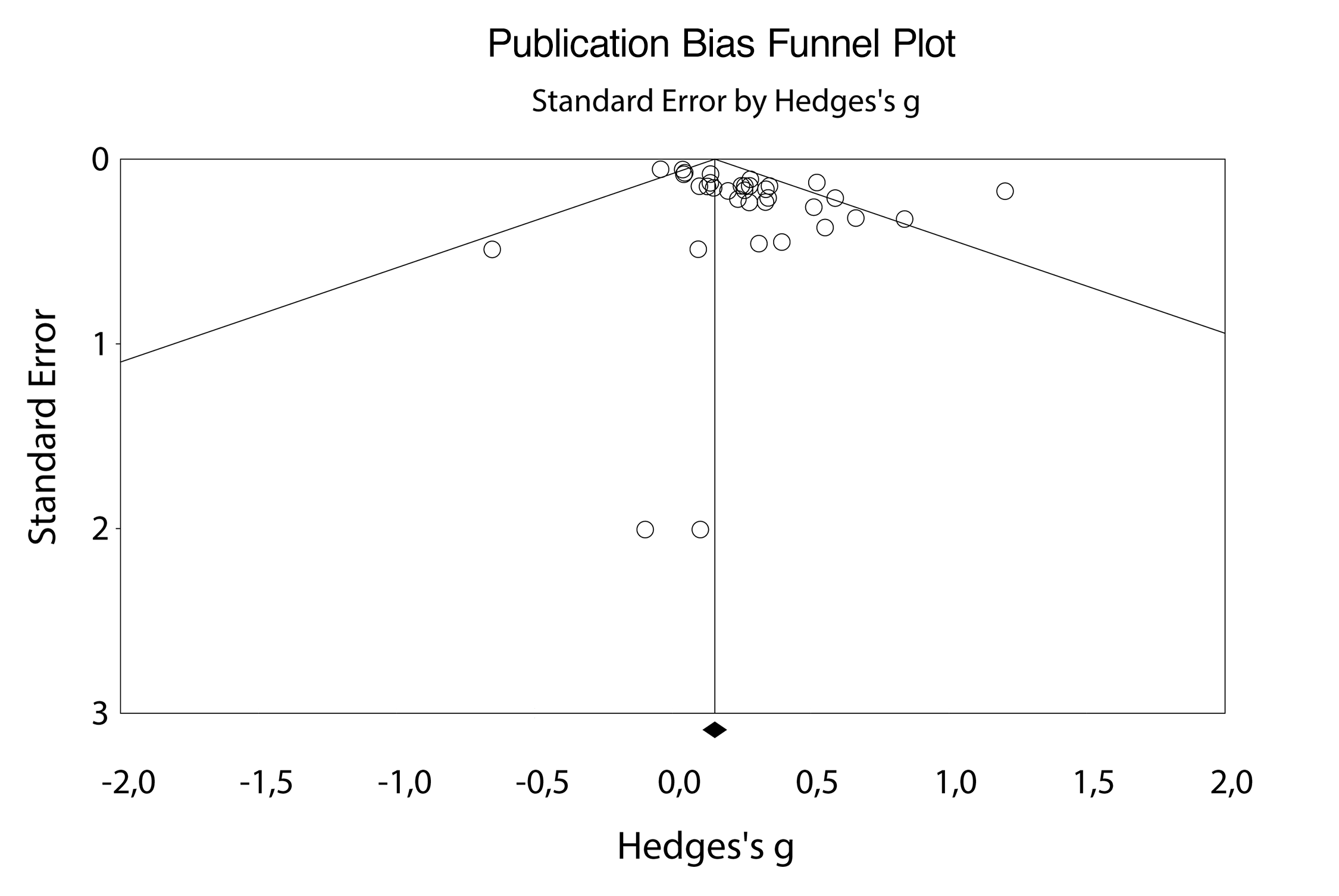

Supplement: S1 Fig — (TIF) [file pmed.1002714.s010.tif]
